# DeepMapi: a Fully Automatic Registration Method for Mesoscopic Optical Brain Images Using Convolutional Neural Networks

**DOI:** 10.1007/s12021-020-09483-7

**Published:** 2020-08-04

**Authors:** Hong Ni, Zhao Feng, Yue Guan, Xueyan Jia, Wu Chen, Tao Jiang, Qiuyuan Zhong, Jing Yuan, Miao Ren, Xiangning Li, Hui Gong, Qingming Luo, Anan Li

**Affiliations:** 1 Britton Chance Center for Biomedical Photonics, Wuhan National Laboratory for Optoelectronics, MoE Key Laboratory for Biomedical Photonics, School of Engineering Sciences, Huazhong University of Science and Technology, Wuhan, China; 2 HUST-Suzhou Institute for Brainsmatics, JITRI Institute for Brainsmatics, Suzhou, China; 3 School of Biomedical Engineering, Hainan University, Haikou, China; 4 CAS Center for Excellence in Brain Science and Intelligence Technology, Chinese Academy of Science, Shanghai, China

**Keywords:** Brain image registration, Deep learning, Convolutional neural networks, Mesoscopic optical images

## Abstract

The extreme complexity of mammalian brains requires a comprehensive deconstruction of neuroanatomical structures. Scientists normally use a brain stereotactic atlas to determine the locations of neurons and neuronal circuits. However, different brain images are normally not naturally aligned even when they are imaged with the same setup, let alone under the differing resolutions and dataset sizes used in mesoscopic imaging. As a result, it is difficult to achieve high-throughput automatic registration without manual intervention. Here, we propose a deep learning-based registration method called DeepMapi to predict a deformation field used to register mesoscopic optical images to an atlas. We use a self-feedback strategy to address the problem of imbalanced training sets (sampling at a fixed step size in nonuniform brains of structures and deformations) and use a dual-hierarchical network to capture the large and small deformations. By comparing DeepMapi with other registration methods, we demonstrate its superiority over a set of ground truth images, including both optical and MRI images. DeepMapi achieves fully automatic registration of mesoscopic micro-optical images, even macroscopic MRI datasets, in minutes, with an accuracy comparable to those of manual annotations by anatomists.

**Electronic supplementary material:**

The online version of this article (10.1007/s12021-020-09483-7) contains supplementary material, which is available to authorized users.

## Introduction

Deconstructing fine neural structures at the cellular level is critical in understanding the connections and collaborations of brain networks (Economo et al. 2015; Gong et al. 2016; Huang and Luo 2015; Li et al. 2015; Oh et al. 2014). In biological research, neuroscientists often manually delineate the brain regions and nuclei in which neurons are located (Fürth et al. 2018; Lein et al. 2007; Lin et al. 2018; Osten and Margrie 2013) with the help of a brain stereotactic reference atlas. However, mainly due to the differences in individual animals and the conditions of specific experimental settings, the correspondence of brain structures with an atlas is highly dependent on personal experience and proficiency (Fürth et al. 2018; Jin et al. 2019; Ni et al. 2018). Moreover, the rapid development of neural circuit labeling methods and whole-brain imaging technologies (Gong et al. 2016; Li et al. 2010; Ragan et al. 2012) have resulted in brain images becoming increasingly complicated at the mesoscopic level. Additionally, the advent of the terabyte-scale (TB-scale) mouse brain dataset (Landhuis 2017) has promoted the need for a stable and reliable high-throughput automatic registration method suitable for these large datasets.

Image registration is a fundamental image processing problem (Zitova et al. 2003) in that has been widely studied in brain science (Niedworok et al. 2016). In particular, a series of representative and effective registration algorithms have been developed for macro MRI images (Klein et al. 2009). However, for mesoscopic optical images, different types of neurons exhibit different image characteristics in different brain regions and diverse labeling strategies are applied; therefore, feature-based registration methods are widely used (Fürth et al. 2018; Jin et al. 2019; Ni et al. 2018; Ohnishi et al. 2016). For instance, Ohnishi et al. registered two-dimensional (2D) micro-optical images to an MRI image by manually locating the feature points (Ohnishi et al. 2016). Fürth et al. registered microscopy images to a reference brain atlas by interactively selecting feature points (Fürth et al. 2018). Ni et al. performed three-dimensional (3D) registration of cellular optical images to a standard brain space using a number of manually acquired nuclei regional features (Ni et al. 2018). However, in these schemes, feature acquisition is still heavily dependent on interactive manual methods and experience; moreover, these approaches are typically expensive and are inefficient for addressing large-scale image datasets. Current studies of neural circuits and cell censuses have produced hundreds of whole-brain samples in large-scale, high-throughput projects (Cyranoski 2017); thus, the lack of effective and efficient registration methods seriously limits comparative brain research at the mesoscopic scale.

Artificial intelligence techniques (Szolovits 2019), especially deep learning methods (Litjens et al. 2017), have gradually been applied to increasingly complex registration tasks (Haskins et al. 2019) because of the ability of deep learning models to automatically extract fine-grained features from high-dimensional images hierarchically (Cheng et al. 2018). Due to the lack of sufficient registered ground truth brain images, the initial unsupervised methods serve only as substitutions for statistical similarity metrics during the image registration process (Cheng et al. 2018; Krebs et al. 2017; Wang et al. 2016). However, the subsequent emergence of a spatial transformation network (STN) (Jaderberg et al. 2015) facilitated the embedding of the deformation process into several unsupervised algorithms based on deep learning networks (Balakrishnan et al. 2018; de Vos et al. 2019, 2017; Li and Fan 2017; Sheikhjafari et al. 2018; Zhang 2018). Nonetheless, the effects of unsupervised methods are highly dependent on the image signals in the brains (Fan et al. 2019), and it is difficult to ensure the registration accuracy when large gaps (such as different image modalities, qualities and large deformations) occur between the corresponding datasets. The supervised methods (Cao et al. 2018; Rohé et al. 2017; Yang et al. 2017) are improving along with the increasing number of registration datasets. Among them, fully convolutional network (FCN) (Shelhamer et al. 2017) models are effective predictors of the deformation field. For example, Rohé et al. (2017) and Yang et al. (2017) developed FCN-based methods to predict deformation parameters in their entirety (Hu et al. 2018; Rohé et al. 2017; Stergios et al. 2018) or in partial form (Ding et al. 2017; Xiao et al. 2017; Yang et al. 2016, 2017) form; these methods have been used to perform registration of heart and human brain MRI images. By selecting training sets via a sliding window and predicting individual pixels, other patch-based methods have also achieved satisfactory registrations with CT (Sokooti et al. 2017) or MRI (Cao et al. 2018) images. However, training an FCN is time-consuming and is not applicable for larger 3D image data (Shen et al. 2019a, b). Although patch-based methods overcome this problem, different optically labeled images have different characteristics, and even patch-based methods have difficulty training various samples for each optical image with specific labeling methods. These factors restrict the training process of supervised methods. Therefore, we need to customize a training strategy that can both expand the trainable data and acquire a more accurate model from limited samples.

Here, we present a method, called DeepMapi, which is based on a convolutional neural network (CNN) to predict the deformation field corresponding to each pair of images and used to automatically register mesoscopic micro-optical imaging datasets to a reference atlas. Within DeepMapi, we used a self-feedback training strategy to accurately capture training samples of varying difficulty levels for “special training”. This strategy is able to obtain a better model from a limited training set. Additionally, inspired by data distillation (Radosavovic et al. 2018), which automatically generates training labels to significantly improve model performance, we design a dual-hierarchical training strategy to improve the model’s applicability to both large and small deformations. By combining these two strategies, DeepMapi enables fully automated registration of mesoscopic optical images and even macroscopic MRI datasets while achieving accuracy levels comparable to those of manual registrations by anatomical experts.

## Methods

### Overview

The goal of 3D brain image registration is to match a moving image to a reference image by transformations to achieve anatomical correspondence (Zitova et al. 2003). The optimal transformations can be obtained through multiple iterations (Eq. 1 and 2).

1 $$ \mathrm{F}=M\circ \phi, $$

2 $$ \hat{\phi}=\arg\ {\min}_{\phi }S\left(\phi; F,M\right)+R\left(\phi \right), $$

where F is the reference image, M is the moving image, S is the similarity metric, φ represents the transformation parameters and R is a regularization term. Here, we focus primarily on nonlinear registration and seek to obtain the nonlinear parameter (φ) automatically and accurately.

**Fig. 1:**
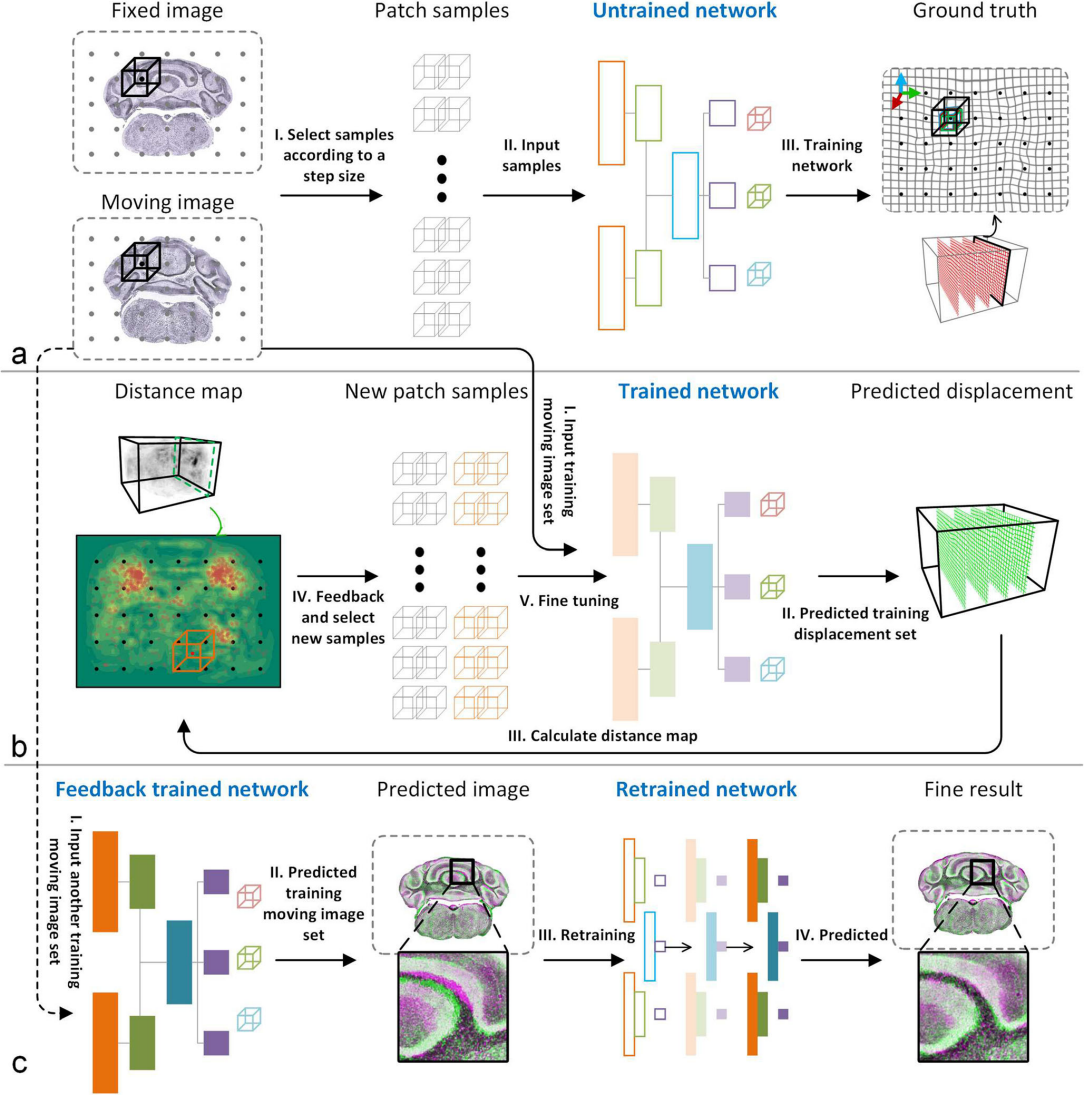
The complete workflow for DeepMapi registration. **a** In the training stage, the training samples are selected according to a fixed step size and window size. These are input into the convolutional neural network to obtain the pretrained model. **b** The first level corrects the large deformations in the brain. Then, the self-feedback training stage reselects samples based on the distance map to fine-tune the previously trained model. **c** In the second level, the prediction results obtained from the first level are used as training sets to correct the remaining small deformations and obtain the fine registration results. Each step is marked with Roman numerals

In this paper, we customize the deep learning method based on a patch-based network (Fig. 1), which seeks to train a better model from a limited number of samples that can adapt to both large and small deformations. Then, we predict the deformation parameter (φ) automatically from pairs of input brain images.

First, we need to choose a reference image and manually register it accurately to the standard brain space. This shifts the goal from how to register other datasets to the reference image. Second, we train a patch-based network (Fig. 1a) and then present a self-feedback strategy that allows us to improve the model’s performance by adjusting the proportion of training samples (Fig. 1b). Finally, a dual-hierarchical CNN training strategy is designed in which the first network (Fig. 1ab) is used to learn the large deformations in the brain samples, and the second network (Fig. 1c) is used to learn the remaining small deformations, further fine-tuning the uncorrected parts. All the animal experiments followed procedures approved by the Institutional Animal Ethics Committee of Huazhong University of Science and Technology, and this study was also specifically approved by that ethics committee. All the methods were conducted in accordance with the relevant guidelines and legislation. We present the details in the following sections.

### Data Sources

1) Source of optical imaging datasets: We obtained 23 datasets of mouse brain images to verify DeepMapi. The datasets were captured by the fMOST (Gong et al. 2016) system, which outputs resin-embedded dual-color labeled C57BL/6 J mouse whole brain datasets. Generally, the resin-embedded samples introduce additional deformations (Gong et al. 2016). The colocalized fluorescent-labeled neurons and counterstained cell bodies dataset in the brain-wide images are acquired at a 2 μm axial resolution and a 0.32 μm horizontal resolution. We adopt the propidium iodide (PI) channels of the datasets during the experiments.

2) Reference brain atlas space: We choose the common coordinate framework (Allen CCF v3) (Dong 2008; Goldowitz 2010; Kuan et al. 2015) from the Allen Institute as the standard brain space. This standard represents an average brain obtained by the continuous registration of 1657 STP datasets and it provides well-segmented brain region annotation files. Four resolution levels (100 μm, 50 μm, 25 μm, and 10 μm) are available (http://download.alleninstitute.org/informatics-archive/current-release/mouse_ccf/annotation/).

3) The temporary reference dataset (fixed image): We selected one brain dataset from the abovementioned optical images and aligned the other datasets to this fixed image.

4) Division of training and validation data and data augmentation: To learn the large and small deformations individually, we classify and augment the remaining 22 datasets to obtain more training sets, which prevents overfitting. These datasets are prealigned to the standard brain space by an automatic linear registration algorithm. Finally, we apply a histogram-matching operation to the datasets using a fixed image to normalize their gray values to values in the range 0–1.

Train_data1: This group includes 10 sets of datasets. The images are isotropically downsampled to a 25 μm resolution (size: 456 × 360 × 528 pixel^3^) and manually registered to the fixed image (BrainsMapi (Ni et al. 2018), achieves good registration results for optical images by segmenting and mapping the regional features). Then, the obtained deformations are shifted once in the negative direction (-Warp) and twice in the positive direction (2*Warp) (Supplementary Fig. 1). These new deformations are applied to the moving images to augment the data. These augmented datasets are used when training the first level, which is a coarse registration process that learns the initial large deformations in the brain samples.

Train_data2: This group includes 7 sets of datasets. These images are also isotropically downsampled to a 25 μm resolution (size: 456 × 360 × 528 pixel^3^). First, we use the trained model from the first level to predict and deform Train_data2. Second, the warped results are registered to the fixed image by BrainsMapi to correct remaining deformations. Finally, the obtained deformations are half-shifted (1/2*Warp) (Supplementary Fig. 1) and applied to the moving image to augment the data. These samples are used during the second-level training, which is a fine registration process that learns the remaining small deformations in the brain samples.

Validation_data: This group includes 5 sets of datasets used to verify the accuracy of the proposed method. These data are also processed using the BrainMapi method.

### Registration of Fixed Image to Standard Brain Space

Since the micro-optical images and reference brain atlas are completely different modal datasets, it is very difficult to map them directly. Therefore, we need to select a good quality micro-optical imaging data as a temporary reference to reduce the modality and deformation differences and then use the BrainsMapi method to register the images to the standard brain space. Subsequently, we can automatically register other newly input datasets to indirectly achieve registration to the standard brain space.

We complete the registration of the fixed image to the Allen CCF v3 brain space using the BrainsMapi method. Supplementary Fig. 2 shows some examples of BrainsMapi’s good registration results.

### The Self-Feedback Strategy

In deep learning image classification or segmentation tasks, the number of training samples is generally the same for each class, and the samples are randomly distributed. In contrast, brain image registration is relatively complicated. First, it is not a simple classification problem. Second, the brain sample deformations are nonuniform across the entire brain range, and the degrees of deformation of different anatomical structures are also different. For regions with large deformations or complex structures, sampling at a random or a fixed step size (Fig. 1a, black dot) will not guarantee that the complex deformations will be fully learned. Therefore, it is necessary to design a new sample selection strategy to adjust the samples during the training process and obtain a better model.

The self-feedback strategy is based on the predicted results from a pretrained model trained at a fixed step size. We predict the training set using this model and acquire the predicted displacement field (P); and then, we calculate the distance errors between the true displacement field (G) and the predicted displacement field (P) to obtain a distance map (Eq. 3). The distance map is presented in both 3D and 2D (Fig. 1b, distance map), where the darker areas indicate larger differences between the predicted and true values. We find that prediction deformation fields with large values or complex structures, such as the cerebellum (CB), are fairly error-prone (Fig. 1a, moving image). Hence, we selectively create more training samples from these regions indicated by the yellow rendering of the distance map, for focused training (Fig. 1b, red dots). Next, we calculate the value of each distance from 1-n, where n is the max distance in DMap and C is a counting function (Eq. 4) that counts the frequency of each distance. Then, the samples are reselected by using Eq. 5 to determine the number of samples that needs to be selected for each distance. Here, S refers to the number of training samples selected for each distance, and A refers to the number of additional samples selected during the self-feedback stage. α is a tunable parameter whose value can be 1/2, 1/4, 1/8, 1/16, etc., based on a square root operation. The smaller the value of α is, the more balanced the samples selected from the distance map are.

3 $$ {DMap}_{(v)}=\left\Vert \begin{array}{c}{G}_{x(v)}-{P}_{x(v)}\\ {}{G}_{y(v)}-{P}_{y(v)}\\ {}{G}_{z(v)}-{P}_{z(v)}\end{array}\right\Vert, $$

4 $$ \mathrm{N}(i)=C\left( Dmap,i\right), i\epsilon \left[1,n\right], $$

5 $$ \mathrm{S}(i)=\frac{{\left(\frac{1}{N(i)}\right)}^{\alpha }}{\sum_{i=1}^n{\left(\frac{1}{N(i)}\right)}^{\alpha }}\ast A, i\epsilon \left[1,n\right]. $$

To ensure that the trained model is not biased, we retain the older samples selected at a fixed step size and use all the samples to fine-tune the previously trained model.

### The Dual-Hierarchical Training Network

The implementation is based on dual inputs of 64 × 64 × 64 image patches that correspond to the local small blocks extracted from the fixed and moving images, while the outputs are three 9 × 9 × 9 deformations in the x, y and z directions in the center of the blocks. Unlike a typical patch-based network, which predicts only a single value, we increase the output size slightly to improve the prediction efficiency.

The gray area in Supplementary Fig. 3 shows the network architecture of DeepMapi. Initially, we input two image blocks from the fixed and moving images and pass them through an InputConv unit independently. The InputConv unit consists of a convolution layer (kernel: 5 × 5 × 5, stride: 1 × 1 × 1, padding: 2 × 2 × 2) and a PReLU activation layer (Ketkar 2017). Next, the learned features pass through three Encoder units. Each Encoder unit is composed of a convolution layer (kernel: 3 × 3 × 3, stride: 1 × 1 × 1, padding: 1 × 1 × 1), a PReLU activation layer and a pooling layer (kernel: 3 × 3 × 3, stride: 2 × 2 × 2, padding: 1 × 1 × 1). The number of feature maps doubles after each Encoder unit, and we concatenate the feature maps from the two branches. Then, Decoder units are used to process the three output branches, which correspond to the deformations in the x, y and z directions. Each branch is composed of three consecutive Decoder units and one OutConn unit. A Decoder unit is the same as an Encoder unit, while the specific purpose of the OutConn unit is to transform the output vector of length 729 into the 9 × 9 × 9 output. We also apply dropout to prevent overfitting during training. The location is indicated in the red box in Supplementary Fig. 3. Moreover, since this is a patch-based method, we can obtain many training samples, which also helps avoid overfitting.

We use Train_data1 to train the network (Fig. 1a) and fine-tune the pretrained model using the self-feedback strategy (Fig. 1b). Based on the trained model, Train_data2 is predicted, and the registration results are obtained at the first level. Then, we use BrainsMapi to map the registered Train_data2 to the fixed image and perform the data augmentation. The new training set is used to train another new model to achieve the fine registration results (Fig. 1c, fine result), and the self-feedback strategy is used during the retraining stage (Fig. 1c, III, retraining). Both levels use the same variant of the patch-based network, but correspond to different datasets with large and small deformations, respectively. In particular, the small deformation datasets are adaptively derived from the first-level model.

### Implementation

(1) Training: We use PyTorch (Ketkar 2017) to implement our method and train a total of two models. In the first model, the input window size is 64, the step size is 32, the output size is 9, the batch size is 64, and the number of feature maps in the first layer is 4. The training set for the first level is Train_data1. The training parameters of the second level are consistent with those of the first level, but the corresponding training set is Train_data2. In addition, we reset the step size to 48 and add approximately 2000 new pairs of samples per set of datasets, as discussed in the feedback training section. The learning rate for both models is 0.0001, and the number of iterations is 200. We use L1loss (Yang et al. 2017) as the loss function and Adam (Ketkar 2017) as the optimization algorithm.

(2) Prediction: We use a 64 × 64 × 64 sliding window and 9 × 9 × 9 steps to traverse the fixed and moving images. The obtained image blocks are input into the model and used predict the results. We add the prediction results to an empty 3D matrix based on the corresponding coordinates, as shown in Eq. 6, and obtain the temporary displacement field, φ_temp.

6 $$ {\phi}_{temp}={\phi}_{(0)}+{\phi}_{id}, $$

7 $$ W={W}_{(0)}+{W}_{id}, $$

8 $$ \upphi =\frac{\phi_{temp}}{W}. $$

Simultaneously, we use an empty 3D matrix to record the number of superpositions (W) for each voxel (Eq. 7). By dividing these two matrices, we can obtain the final deformation field φ (Eq. 8). Additionally, we perform mean filtering on the deformation field to smooth it.

The prediction process also involves two levels. Suppose that the result obtained from the first level is calculated by the function *G*(*x*), and the result of the second level is calculated by the function *H*(*x*); then, the final output result will be *M* ’ = *H*(*G*(*M*)). That is, the output of the first level is used as the input to the second level to obtain the final prediction results.

### Registration Methods Used for Comparisons

Affine: We use the ANTs tool (Klein et al. 2009) to perform linear registration, including rotation, scaling, translation, and shear transformations. The linear registration results provides the initial values for a nonlinear registration.

LD-Demons (Vercauteren et al. 2008): A nonlinear registration algorithm based on the log-domain demons diffeomorphic and symmetric local correlation coefficient (LCC) similarity metrics to perform the nonlinear transformation.

SyN (Avants et al. 2008): A nonlinear registration algorithm based on the symmetric diffeomorphic normalization transformation (ANTs tool, version 2.x) and several similarity metrics.

QuickSilver (Yang et al. 2017): A nonlinear registration algorithm based on a convolutional neural network to predict the momentum of the corresponding image pairs in a blockwise FCN method that is then converted to displacement to achieve nonlinear registration.

VNet (Milletari et al. 2016): A 3D FCN method commonly used in medical image segmentation. We set the number of input channels to two and the number of output channels to three for image registration applications.

Here, we mainly focus on comparing DeepMapi with the above traditional and supervised deep learning methods. Moreover, we also compare DeepMapi with several recent unsupervised deep learning methods, including VoxelMorph (Balakrishnan et al. 2019; Dalca et al. 2019), AVSM (Shen et al. 2019a) and RDMM (Shen et al. 2019b). In fact, due to the larger data size and lack of training sets (one brain is one sample), these unsupervised methods are not particularly suitable for optical brain datasets. Hence, we first downsample the datasets to the size required by these methods and then conduct training.

### Quantitative Evaluation Method

We choose two measurements to comprehensively assess DeepMapi. The first is L1loss, which is the absolute error. This relationship can be represented by a correlation plot. We use the two axes to represent the displacements of the true deformation and the predicted deformation and plot them in the form of a dot plot.

Another metric commonly used in image registration is the Dice score (Dice 1945), which reflects the voxel overlap. The higher the Dice score is, the higher the overlap rate is, which means more accurate results. We use the registration results of the BrainsMapi method as the gold standard for the Validation_data datasets to quantitatively assess DeepMapi.

We also evaluate the diffeomorphic property of DeepMapi displacement. Because tissue folding is anatomically impossible, we evaluated the topology of the obtained displacement field by using the *Jacobian* determinant (de Vos et al. 2019). The *Jacobian* determinant (Eq. 9) captures the *Jacobian* for every voxel *p* in the displacement field:

9 $$ \mathit{\det}\left(J\left(i,j,k\right)\right)=\left|\begin{array}{ccc}\frac{\partial_i}{\partial_x}& \frac{\partial_j}{\partial_x}& \frac{\partial_k}{\partial_x}\\ {}\frac{\partial_i}{\partial_y}& \frac{\partial_j}{\partial_y}& \frac{\partial_k}{\partial_y}\\ {}\frac{\partial_i}{\partial_z}& \frac{\partial_j}{\partial_z}& \frac{\partial_k}{\partial_z}\end{array}\right|. $$

A *Jacobian* > 0 indicates that the local deformation is diffeomorphic, both invertible and orientation-preserving, while a *Jacobian* ≤ 0 indicates a location where folding occurs (de Vos et al. 2019).

### Computing Environment

We used two different computing platforms to train and test the proposed method, a graphic workstation and a GPU-equipped server. The graphic workstation is configured with an Intel (R) Xeon(R) E5–2699 v4 CPU @ 2.20 GHz with 1 TB of memory, while the GPU server equipped with a Quad Tesla-V100 GPU card.

## Results

### Performance Analysis and Optimization of Neural Network

Limited by GPU memory capacity, we perform prediction block by block, which involves two strategies, the FCN and the patch-based methods. We design a patch-based deep neural network and five other models (Supplementary Fig. 3, Supplementary Table 1) to optimize the model selection and patch sizes. The FCN models are designed following the QuickSilver (Yang et al. 2017) method by adding residual modules similar to UNet (Ronneberger et al. 2015). The FCN models are tested by fixing the input and output sizes (16, 32 and 64). The patch-based models are based on the model proposed in the Methods section; we test these models under a variety of input sizes (16, 32 and 64) and output sizes (3, 5 and 9). For detailed information on the model design and training parameter settings, please refer to the Methods section and Supplementary Table 1.

**Fig. 2:**
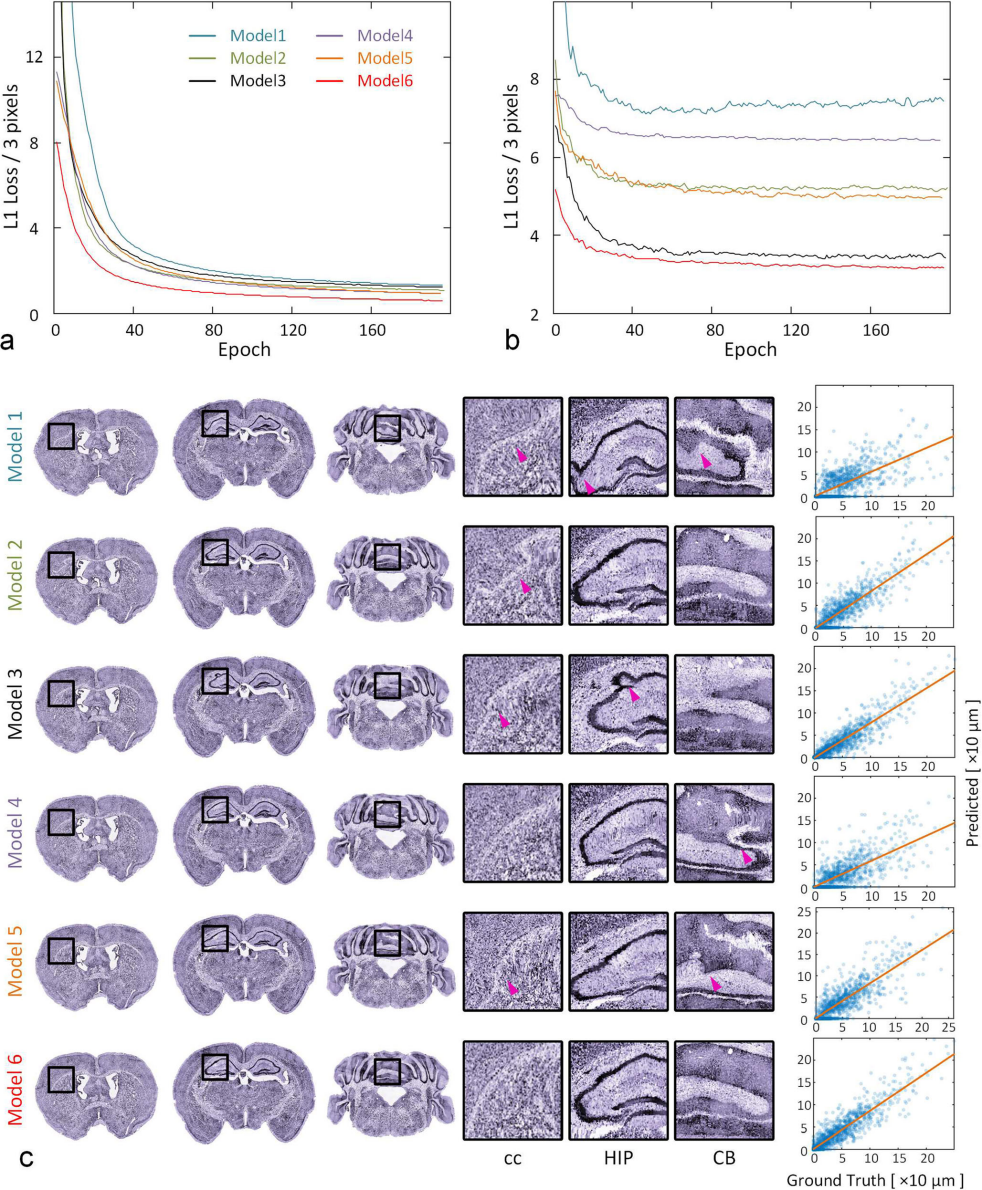
Determine the most appropriate model and its parameters for DeepMapi. Loss curves of the training set (**a**) and validation set (**b**). The ordinate is the L1loss/3 pixels, and the abscissa is the epoch number. The curves for the different models are marked with different colors corresponding to the line marker. **c** The visualized prediction results (left, coronal planes) and correlation plots (right, dot plots) of six models (denoted as Models 1–6). Each model is represented by three coronal planes, and the enlarged views of local regions and nuclei shown on the right correspond to the black boxes in the left-hand images

Figure 2ab shows both the training and validation loss curves during training. A smaller loss value indicates more accurate registration results. We find that the loss value of Model 6 is the lowest for both the training and validation sets (Fig. 2ab, red line, Supplementary Table 2), and that generally, the accuracies of the patch-based models are better than those of the FCN models with the same input size (Fig. 2b). We use three coronal planes to check the registration accuracy of the six models (Fig. 2c) and illustrate some interesting regions, such as the corpus callosum (cc), hippocampal region (HIP) and CB. We find that Models 1–5 show distortions in some areas (Fig. 2c, purple arrow) and that the Model 6 is better than the other five models.

Additionally, the right panel of Fig. 2c shows correlation plots of the six models. The distance from the points to the midline indicates the accuracy, and darker colors indicate more concentrated points and better continuity of the deformation field. Regardless of whether the model is an FCN (Fig. 2c, Model 1–3) or patch-based (Fig. 2c, Model 4–6), the predicted results become more accurate as the input patch size increases. Moreover, the points of Model 6 are closer to the midline and more aggregated than those of Model 3, which suggests that the accuracy and continuity of Model 6 are the best. To prove this, we randomly selected 750 points (150 points from each of the five datasets) from their correlation plots and then calculated the absolute values of the predicted and true values. Subsequently, we performed a paired statistical analysis. The statistics indicate that the results of Model 6 are significantly better than those of the other five models (Wilcoxon signed-rank test, *P* < 0.05; from 5 datasets, 150 points/set) (Supplementary Table 3). We also provide quantitative results of 10 brain regions in the whole brain for six models (Supplementary Table 4) using Dice scores and then performed a paired statistical analysis. The statistics show that the Dice scores of Model 6 are significantly better than those of the other four models (Wilcoxon signed-rank test, model 1–2 and model 4–5, P < 0.05; from 5 datasets, 10 regions/set) and that there are no significant differences between Model 6 and Model 3 (Wilcoxon signed-rank test, *P* = 0.080; from 5 datasets, 10 regions/set) (Supplementary Table 3).

It is also worth mentioning the comparisons of training and prediction times for the different models (Supplementary Table 2). Although the FCN provides faster results, its accuracy is lower. Among the patch-based methods, the prediction time of Model 6 is the fastest; it requires only approximately 5 mins, which is acceptable. Moreover, regarding training time, the number of parameters in the FCN model is much larger and the network architecture is more complicated; therefore, training the FCN takes longer (e.g., Model 3, required approximately 2 weeks), which makes it inefficient during training and testing.

### Effectiveness of the Self-Feedback Strategy

**Fig. 3:**
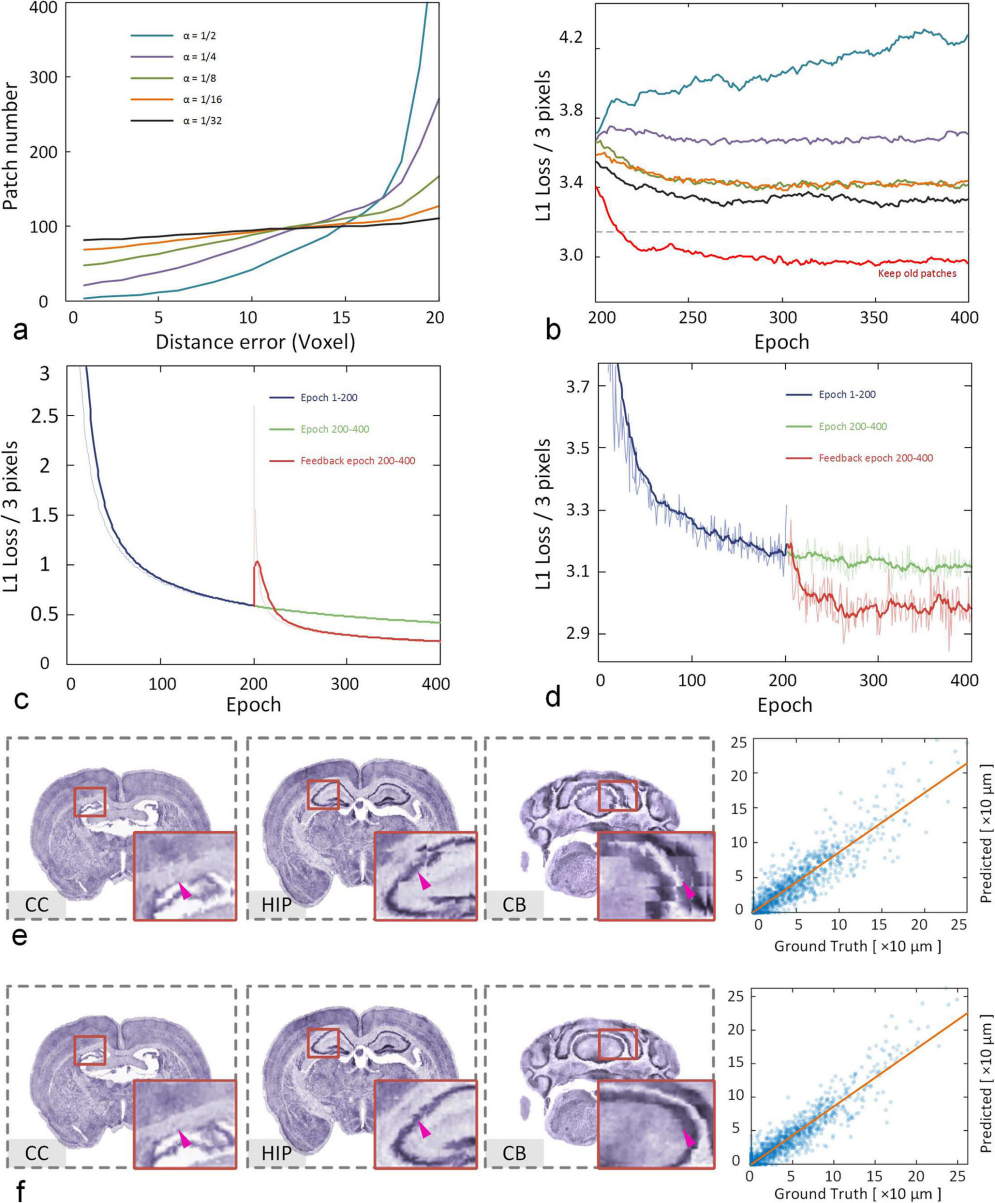
The performance of the self-feedback strategy under different parameter settings. **a** The change in the number of samples for each distance error under different α values. The smaller α is, the more balanced the number becomes. **b** The loss curves of different α values corresponding to (**a**). The red line shows the results of retaining the old patches, and the gray dotted line is the loss value of the pretrained model. The loss curves of the training set (**c**) and validation set (**d**) with (red line) or without (green line) the self-feedback strategy are shown. The visualized prediction results (left, coronal planes) and correlation plots (right, dot plots) before (**e**) and after (**f**) the self-feedback strategy are shown. The registration results are presented by the three coronal planes and the corresponding enlarged views of the local regions in the bottom right corner

This section mainly analyzes the effectiveness of the self-feedback strategy. According to the description in the Methods section, it is necessary to obtain a pretrained model using a specific step size in the first 200 epochs. Based on this model, different α values of 1/2, 1/4, 1/8, 1/16, and 1/32 are selected for the fine-tuning model and testing the performance. As the value of α decreases, the samples selected from the distance map become more balanced (Fig. 3a). Based on the new samples, the pretrained model is fine-tuned for another 200 epochs but the previous samples are not added. As the samples became more balanced, the validation loss continued to decrease (Fig. 3b). However, the loss value is still higher than the loss of training at a fixed step size (Fig. 3b, gray dotted line). But if we choose 1/32 for the α value and retrain the previous samples, the loss value decreases significantly (Fig. 3b, red line).

It is also worth mentioning how to choose the appropriate number of epochs to improve the training efficiency. First, we need to carefully watch the loss changes of the training and validation sets during training. Especially for the validation loss, when we trained for 100 ~ 200 epochs at a fixed step size, the loss value dropped very slowly (Fig. 3d). Hence, we can set the number of epochs to about 200. Second, when an additional 50 ~ 100 epochs are used for training during the self-feedback stage, the loss value of the validation sets starts to fluctuate and no longer declines (Fig. 3d); thus, we can also set a large number of epochs for observation. Hence, we adopted 200 epochs for both training processes.

We compare the trends of the training and validation loss values before and after self-feedback (Fig. 3 cd). As the training time increases, the loss decreases gradually, but its rate of decline slows (Fig. 3 cd, blue line). Compared to continuous training without a feedback strategy (Fig. 3 cd, green line), both the training and validation loss decrease after applying self-feedback (Fig. 3 cd, red line). The direct benefit of this mechanism is that both the accuracy and continuity of the results improve, as reflected in the correlation plots (Fig. 3ef, right). The dots are darker, more focused and closer to the midline after applying self-feedback (Fig. 3f, right). We randomly select 750 points (150 points from each of the five datasets) from their correlation plots and then calculated the absolute values of the predicted and true values; then, we performed a paired statistical analysis. The statistics show that after applying self-feedback, the results are significantly better than those before self-feedback (Wilcoxon signed-rank test, *P* = 0.001; from 5 datasets, 150 points/set).

In addition, we selected several coronal planes from the entire brain to check the registration effects before and after self-feedback (Fig. 3ef). Intuitively, mean filtering is not applied to the displacement field. According to the enlarged views of several representative nuclei (cc, HIP, CB), we find that the registered images have obvious stitching artifacts before applying self-feedback (Fig. 3e) but that these artefacts are greatly reduced after self-feedback (Fig. 3f, purple arrow). This result suggests that the feedback mechanism increases the consistency of the deformation fields.

For quantitative evaluation of the self-feedback strategy, we computed the Dice scores for 10 brain regions in the whole brain before and after self-feedback (Supplementary Fig. 4, Supplementary Table 5). The results show that the feedback strategy effectively increases the registration accuracy of these brain regions. We performed a paired statistical analysis on the Dice scores and found that the registration accuracy after self-feedback was significantly better than that before self-feedback (Wilcoxon signed-rank test, *P* = 2.231e-7; from 5 datasets, 10 regions/set).

### Effectiveness of the Dual-Hierarchical Strategy

Inspired by the data distillation process (Radosavovic et al. 2018), which generates training labels automatically, a coarse deformation model is obtained at the first level; subsequently, the coarse model is applied to other datasets to derive small deformation training sets to improve the adaptability of the model to large and small deformations. To accomplish this, we design a dual-hierarchical training network that learns the large deformations first, followed by the remaining smaller deformations.

**Fig. 4:**
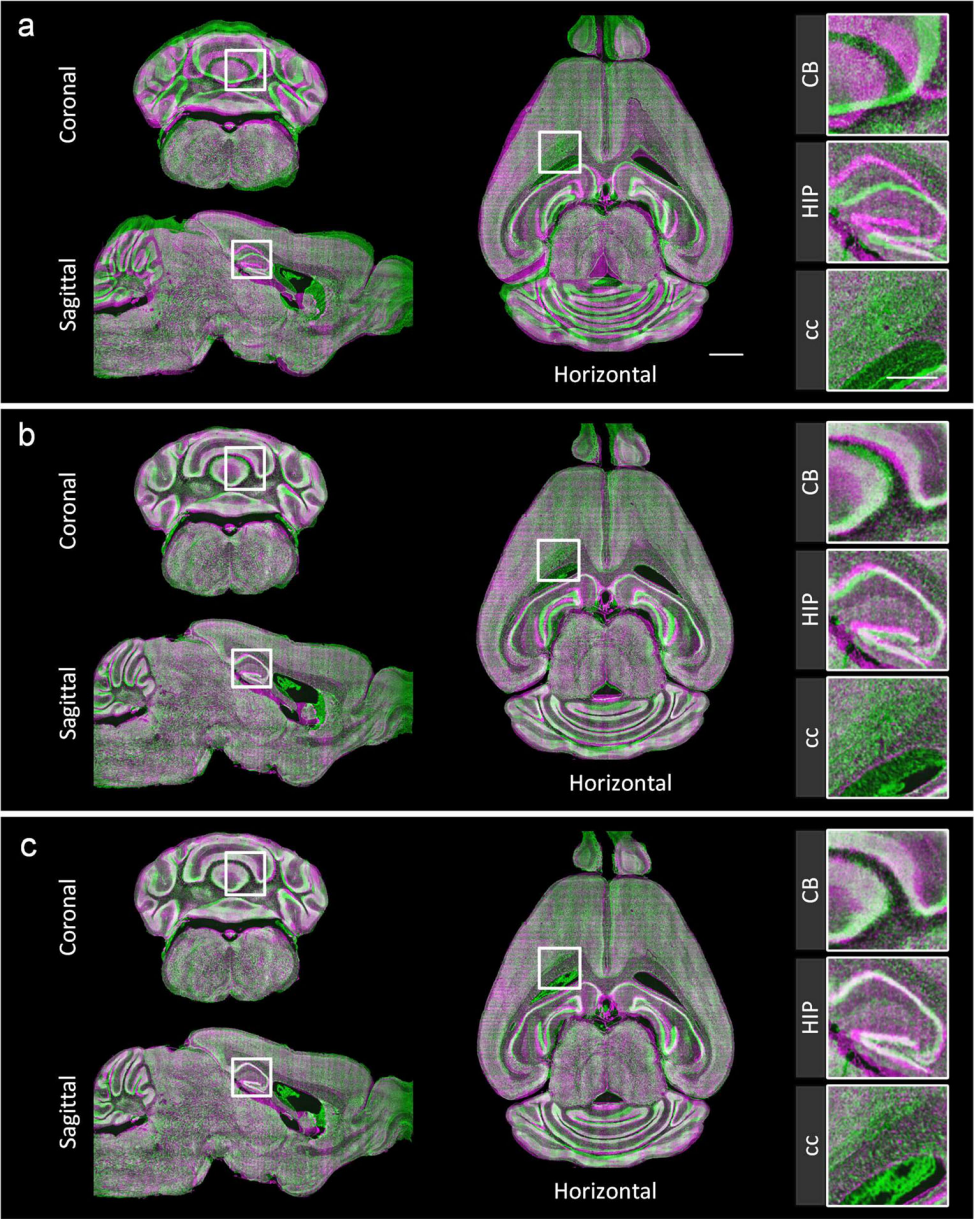
The performance of the dual-hierarchical strategy. The reconstructions of sagittal, horizontal and coronal planes of the linear (**a**), first level (**b**), and second level (**c**) registration results are the fused results of the fixed image (purple) and the moving image after registration (green). The projection thickness is 10 μm. The enlarged views of the local regions shown on the right correspond to the white boxes on the left. Scale bars: 1 mm, detail 0.5 mm

We analyze and compare the registration results of these two levels and present the fusion of the coronal, sagittal and horizontal planes of the registered and fixed images before and after registration (Fig. 4, purple, fixed image; green, moving image). The comparison of the linear registration results (Fig. 4a) shows that the coarse registration in the first level is able to correct the large deformations (Fig. 4b) while the fine registration in the second level further adjusts the remaining small deformations (Fig. 4c). Enlarged views of several nuclei (CB, HIP, cc) are also presented; and the registration results of these nuclei are well fused after the two registration levels (Fig. 4).

To evaluate the dual-hierarchical strategy, we also conducted a quantitative assessment of the registration results at both levels. Because the second level corrects the small deformations, we selected several smaller brain regions and nuclei from the whole brain to evaluate DeepMapi. The Dice scores of these brain regions and nuclei improve after the second level registration (Supplementary Fig. 5, Supplementary Table 6). To prove this, we performed a paired statistical analysis on the Dice scores which showed that the registration accuracy of the second level registration is significantly better than that of the first level registration (Wilcoxon signed-rank test, *P* = 5.837e-6; from 5 datasets, 10 regions/set).

### Comparison of Different Registration Methods to the Reference Brain Atlas

Neuroscientists are generally more concerned with whether they can achieve an accurate registration to the standard brain space. Using the proposed method, the results of registration to the reference brain atlas are presented. These results were acquired by four continuous parameter transformations: (1) the linear parameter, (2) the coarse registration in the first level (nonlinear parameter), (3) the fine registration in the second level (nonlinear parameter), and (4) the BrainsMapi method (Ni et al. 2018) (the fixed image to the brain atlas nonlinear parameter). As a result, we can automatically register brains to the standard brain atlas and obtain brain spatial localization information after the above four transformations.

We registered the testing dataset to the Allen CCF v3 standard brain space for demonstration purposes and compare the registration effects of the proposed method with those of other traditional or deep learning methods. In this study, five traditional registration methods and supervised deep learning registration methods were specifically selected for the comparisons (Methods section).

**Fig. 5:**
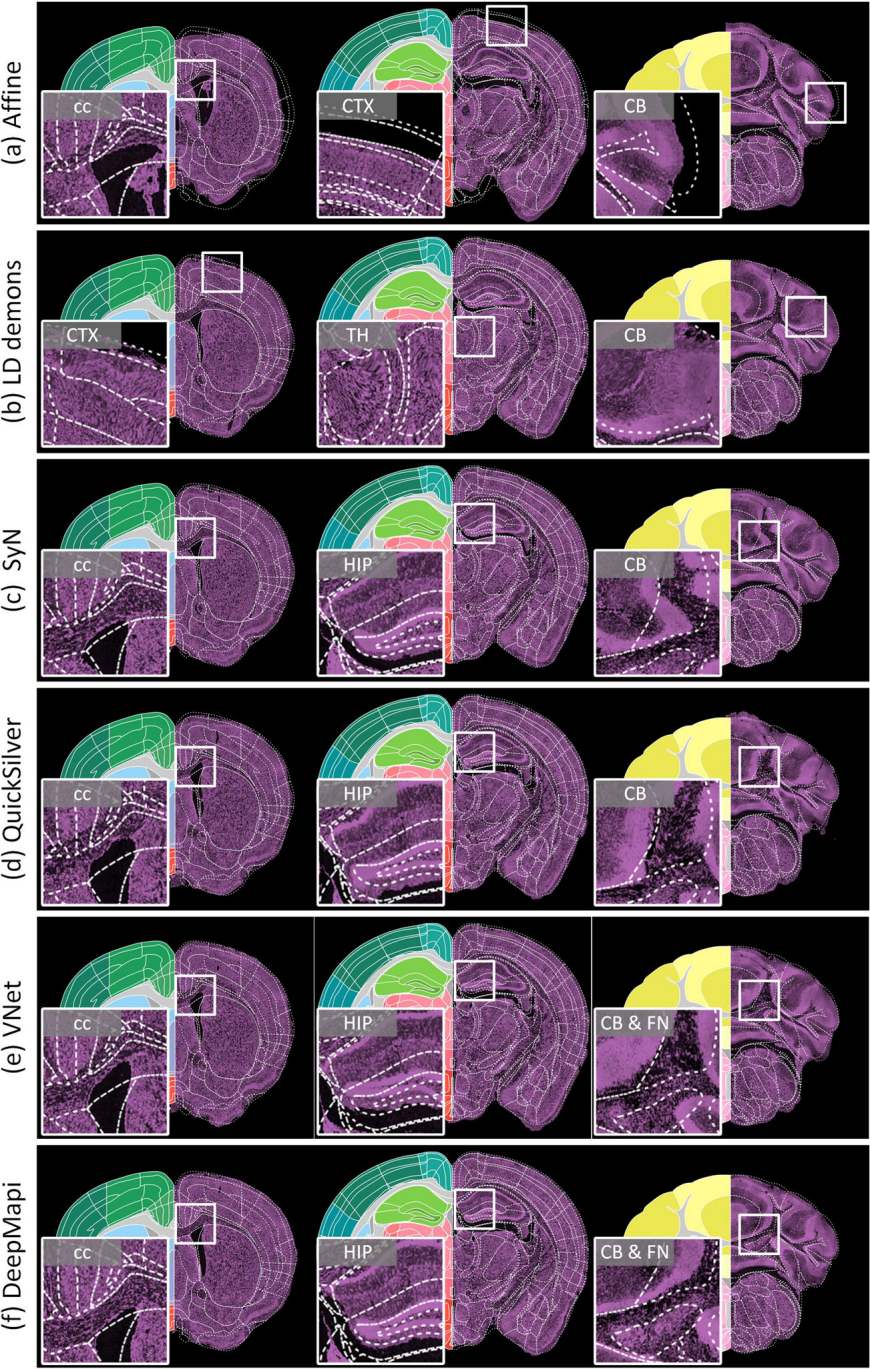
Comparisons of different registration methods. Three coronal sections. The left part of the image is the Allen CCF v3, while the right part of each image shows the white dotted line of the Allen CCF v3 superimposed on the registered image. Enlarged views of the local regions are presented on the left, corresponding to the white boxes on the right. **a** Affine. **b** LD demons. **c** SyN. **d** QuickSilver. **e** VNet. **f** DeepMapi. The region lines is from the Allen CCF v3, © 2004 Allen Institute for Brain Science. Allen Mouse Brain Atlas. Available from: atlas.brain-map.org

Figure 5 shows the effects of the five registration methods and DeepMapi. The left half of each subfigure shows the Allen CCF v3, and the right half shows the outlines of the Allen brain atlas superimposed on the registered images. The enlarged views on the left corresponding to the white box on the right are also presented. The linear registration results clearly result in inaccurate brain contours for several large brain regions (Fig. 5a), including the cc, cerebral cortex (CTX) and CB. The LD-demons (Vercauteren et al. 2008) can map the boundaries of brain regions and some nuclei, such as the texture of cc, HIP and CB, but they are not sufficiently robust across the entire brain and sometimes produce violent deformations, as shown by the enlarged regions (Fig. 5b) of the CTX, thalamus (TH) and CB. The SyN algorithm (Avants et al. 2008) can correct small deformations in some brain regions, such as cc and HIP, but yields inaccurate deformation results for more complex regions with larger deformations, such as the CB regions (Fig. 5c). We also present the registration results using the deep learning registration methods, such as QuickSilver (Yang et al. 2017) (Fig. 5d) and VNet (Milletari et al. 2016) (Fig. 5e). The results of DeepMapi on the cc, HIP, CB and fastigial nucleus (FN) are more accurate than those of the two deep earning methods (Fig. 5f).

**Fig. 6:**
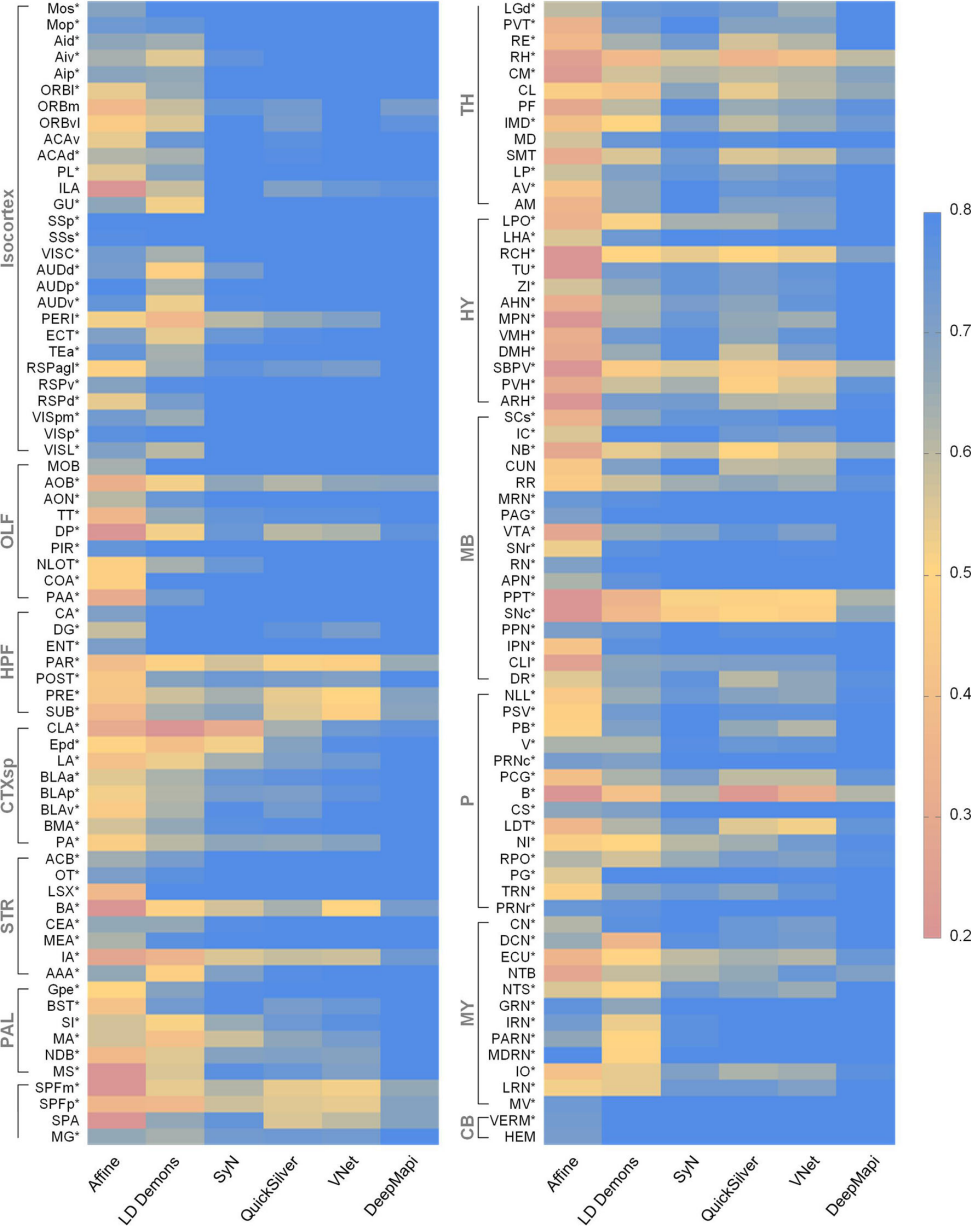
Quantitative evaluations of different registration methods. Heat map of 140 brain regions or nuclei. The applied metric is Dice scores. The closer a color is to red, the lower the registration accuracy is, and the closer a color is to blue, the higher the registration accuracy is. Quantitative evaluations of Affine (0.493 ± 0.200), LD demons (0.637 ± 0.136), SyN (0.756 ± 0.106), QuickSilver (0.728 ± 0.103), VNet (0.740 ± 0.122) and DeepMapi (0.825 ± 0.081) are presented. Additionally, an asterisk (*) is used to mark the regions with the highest DeepMapi values

To conduct a comprehensive assessment, we quantitatively evaluated the registration results of the above five methods and DeepMapi by selecting 5 datasets and 140 brain regions or nuclei from the whole brain. The evaluation covers a total of 11 large brain regions: the isocortex, olfactory areas (OLF), hippocampal formation (HPF), cortical subplate (CTXsp), striatum (STR), pallidum (PAL), TH, hypothalamus (HY), midbrain (MB), pons (P) and medulla (MY). The Dice scores for the 140 regions were calculated using these different registration algorithms and visualized in different colors. The final results are shown as a heat map (Fig. 6). As Fig. 6 shows, the results of the proposed DeepMapi are notably better than those of the other methods. According to the Dice scores listed in Supplementary Table 7, DeepMapi achieves the highest accuracy in most regions.

Moreover, we also present the registration results of three unsupervised methods: VoxelMorph, AVSM and RDMM (Supplementary Fig. 6) and perform a paired statistical analysis with the Dice scores of DeepMapi and other methods. The results prove that DeepMapi is significantly better than the other methods (Wilcoxon signed-rank test, *P* < 0.05; from 5 datasets, 140 regions/set) (Supplementary Table 8).

### Performance Benchmark

**Fig. 7:**
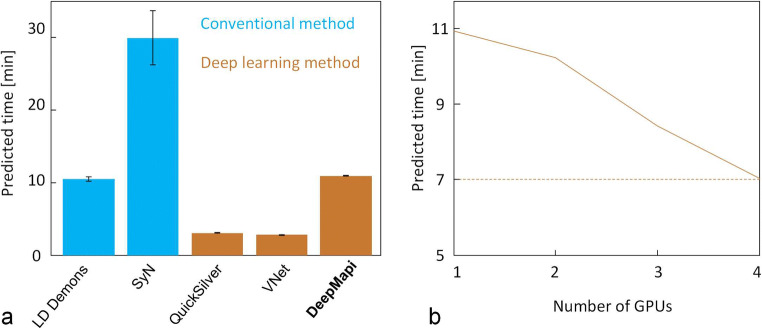
Performance comparisons. **a** The prediction times of different registration methods are presented in the bar chart, where blue indicates the conventional registration methods, and brown indicates the deep learning methods. **b** The graph shows the change in the prediction time of DeepMapi as the number of GPUs increases

We benchmarked the prediction speed of the above four nonlinear registration methods and DeepMapi, and compared the efficiency of DeepMapi on a GPU server (Fig. 7). The results indicate (Fig. 7a) that QuickSilver and VNet provide substantially faster prediction times due to their FCN strategy. Among the traditional registration methods, SyN is the slowest (30 mins), while LD-demons is faster, achieving times similar to those of DeepMapi. In contrast, DeepMapi takes only approximately 10 mins even when running on a single GPU, which is clearly acceptable compared with the traditional SyN method or the manual BrainsMapi method (1 day). In other words, we need only wait 10 mins to achieve accurate and fully automatic registration, while both avoids painstaking manual operations and achieves higher accuracy than previous methods.

We also tested the DeepMapi model using multiple GPUs to predict the results in parallel, which further reduces the prediction time (Fig. 7b) to 7 mins when 4 GPUs are used for prediction.

DeepMapi includes two operations that guarantee the smoothness of the displacement field. The first is soft supervision, which learns the smooth characteristics from the input deformation field. In addition, in post-processing, we perform mean filtering on the deformation field to smooth it. This can be achieved simply by using convolution operations in the neural network (Methods section) to ensure the smoothness of the displacement field. We calculated the *Jacobian* determinant of the 3D displacement field obtained by DeepMapi on the validation sets and found no voxels with negative *Jacobian* values (Supplementary Fig. 7). In addition, we also provided the *Jacobian* determinant for Model 1–5, before/after self-feedback and with/without dual-hierarchical (Supplementary Fig. 8) and found that the smooth characteristics were well learned (zeros voxels with negative values) after the self-feedback and dual-hierarchical strategies.

### Validation Using MRI Datasets

In addition to optical brain images, DeepMapi can also be applied to register MRI datasets. We tested the performance of DeepMapi using public MRI datasets (LONI LPBA40) (Shattuck et al. 2008) by selecting one dataset as a reference, using 30 datasets as training sets, and treating the remaining 9 datasets as validation sets. The gold standard training labels were obtained from the displacement field obtained by the SyN method. Using this setup, we trained a new DeepMapi model on LONI LPBA40.

**Fig. 8:**
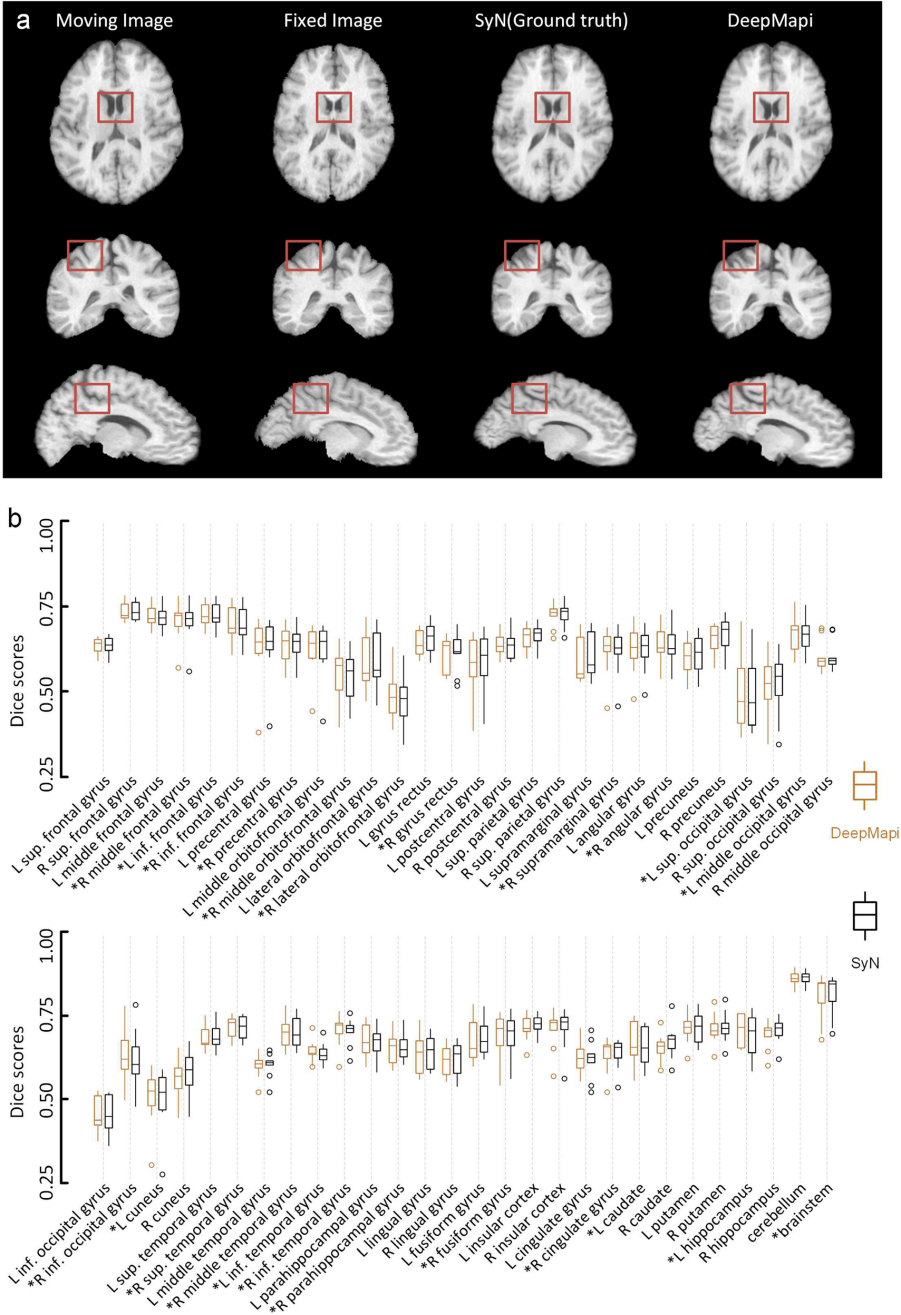
The performance and quantitative assessment of SyN and DeepMapi on the LONI LPBA40 datasets. **a** The registration results of SyN (ground truth) and DeepMapi. Three standard anatomical planes are presented from left to right and the four columns correspond to the moving image, fixed image, SyN registration results and DeepMapi registration results, respectively. The brown boxes represents the same spatial position for the comparisons. **b** Quantitative assessment of 56 brain regions. The Dice scores of nine brain datasets are presented in a box plot. Brown: DeepMapi. Black: SyN. The regions where DeepMapi achieved a higher median value than SyN are marked with an asterisk (*)

In Fig. 8a, we show the moving image, fixed image, SyN registration results and DeepMapi registration results. Clearly, the results of DeepMapi are very similar to the results of the SyN method. Furthermore, we predicted the 9 validation datasets and computed the Dice scores of 56 brain regions. The results are shown as a box plot (Fig. 8b). The accuracy scores of the DeepMapi method and the gold standard (SyN) are almost identical. We performed a paired statistical analysis on the Dice scores of DeepMapi and SyN, grouped the Dice scores by different methods and found no significant differences between DeepMapi and the gold standard (SyN) (Wilcoxon signed-rank test, *P* = 0.191; from 9 datasets, 56 regions/set).

DeepMapi is a fully supervised method. Compared with unsupervised or semisupervised methods, the accuracy of DeepMapi does not exceed the gold standard provided during training, but it does ensure the stability of the registration results which is particularly important. Here, small and uniform gray MRI datasets are used to demonstrate that DeepMapi can learn the features sufficiently and obtain a fine model. The accuracy of the registration results depend heavily on the training sets provided to the network.

## Discussion

The recently developed deep learning registration methods have concentrated on improving the loss function and refining model architectures (Fan et al. 2019; Yang et al. 2017; Zhang 2018); however, for more complex mesoscopic optical imaging datasets, applying such inherent strategies are not particularly effective. Based on the deep learning methods from previous works, we focus on the processes of training sample selection and the training process itself. The result is a fully automatic deep learning registration method for 3D brain image datasets. Our targeted modifications include the following.

1. We design a feedback training strategy that adjusts the weights of the training samples, making it possible to accurately select regions with larger and more complex deformations for additional “special training”. We further compared of the results of the self-feedback strategy by simply sampling more patches with smaller step sizes. We reduced the step size from 32 to 16 by intervals of 4 and found that while *p* value is increased, it was still less than 0.05; moreover, the training time increased sharply to approximately 21 days (Supplementary Table 9). The redundancy in the training samples will greatly reduce the efficiency during application. The results also prove that the proposed self-feedback strategy can exactly solve the contradiction between training efficiency and accuracy.
2. We further employed a hierarchical registration framework that is not only able to solve the small deformation registration problems but is also appropriate for large deformations. The complicated sample preparation process used during in vitro imaging leads the brain sample deformations, which is the main reason why registration is necessary.

Using this method, we built a fully automatic registration method for mesoscopic scale optical images that produces accurate registration results in minutes that are consistent with manual registration. We also demonstrate that DeepMapi generalizes well to other data modes, such as MRI datasets. As long as sufficient training sets are available, we expect DeepMapi to be suitable for registration tasks on other mesoscopic optical image datasets, including zebrafish, monkeys, and even human brains. Additionally, the proposed self-feedback strategy is promising for situations where samples have nonuniform distributions, including tasks such as image denoising and image restoration.

Due to the large variety of application-specific optical labeling methods (Fürth et al. 2018; Gong et al. 2016; Lin et al. 2018) in modern neuroscience, the characteristics of image datasets differ widely; thus, it is difficult to achieve accurate registration based directly on the original signals characteristics. To solve this problem, we used PI-stained cytoarchitectural information (Gong et al. 2016) for registration. In neuroscience research, tissue sections with nucleic acid staining (e.g. PI, DAPI) are often used to localize specific labeled neural circuits (Gong et al. 2016). In fact, we expect our method to be able to register almost all datasets that show the anatomical features of brain regions or nuclei, such as Nissl staining (Li et al. 2010), autofluorescence (Niedworok et al. 2016), and MRI images (Johnson et al. 2010).

Advances in brain sciences and technologies have resulted in the accumulation of large numbers of brain datasets (Landhuis 2017), especially 3D whole brain datasets at the mesoscopic level, which are more conducive to studying relationships between structure and function. These intricate, spatiotemporal and scattered brain datasets could form a comprehensive brain spatial information system if they existed in a common brain space (Boline et al. 2008). Brain registration provides spatial coordinate information to bridge these scattered datasets; however, these massive datasets place high demands on good registration methods in terms of accuracy, automation and speed. The DeepMapi method proposed here is well-matched to these challenges; users can easily execute the program and obtain accurate registration results for a standard brain space in minutes.

## Information Sharing Statement

The datasets and code for demonstrating the method are available at:

http://atlas.brainsmatics.org/a/ni2020.

## Electronic supplementary material

ESM 1 (PDF 1424 kb)
