# Supplementary material for: DeepMapi: a Fully Automatic Registration Method for Mesoscopic Optical Brain Images Using Convolutional Neural Networks
Source: Neuroinformatics. 2020 Aug 4;19(2):267–84. doi: 10.1007/s12021-020-09483-7 (PMC8004526; doi:10.1007/s12021-020-09483-7)
Supplement: Supplementary file 1 — (PDF 1424 kb) [file 12021_2020_9483_MOESM1_ESM.pdf]

Supplementary materials

# DeepMapi: A Fully Automatic Registration Method for Mesoscopic Optical Brain Images Using Convolutional Neural Networks

Hong Ni[1], Zhao Feng[1], Yue Guan[1], Xueyan Jia[2], Wu Chen[1], Tao Jiang[2], Qiuyuan Zhong[1], Jing Yuan[1, 2], Miao Ren[2, 3], Xiangning Li[1, 2], Hui Gong[1, 2, 4], Qingming Luo[1, 2, 3], Anan Li[1, 2, 4,*]

[1] *Britton Chance Center for Biomedical Photonics, Wuhan National Laboratory for Optoelectronics, MoE Key Laboratory for Biomedical Photonics, School of Engineering Sciences, Huazhong University of Science and Technology, Wuhan, China*

[2] *HUST-Suzhou Institute for Brainsmatics, JITRI Institute for Brainsmatics, Suzhou, China*

[3] *School of Biomedical Engineering, Hainan University, Haikou, China*

[4] *CAS Center for Excellence in Brain Science and Intelligence Technology, Chinese Academy of Science, Shanghai, China*

[*] Corresponding author.

E-mail: aali@mail.hust.edu.cn (Li A)

**Supplementary Fig. 1: The registration results used for training and augmentation.**

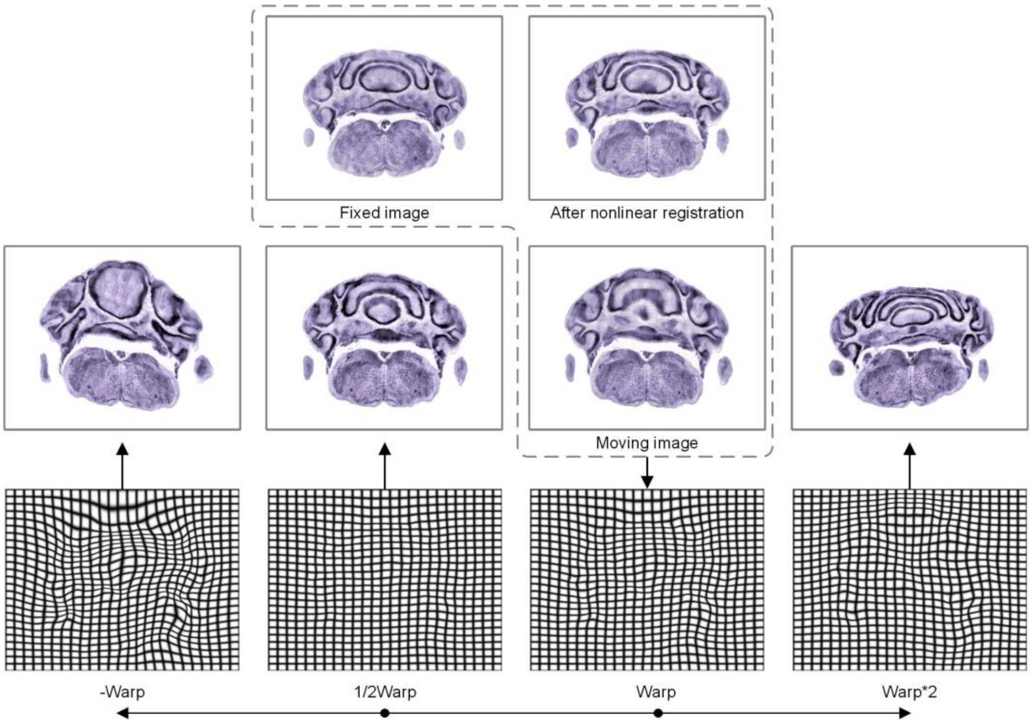


The dashed box shows the registration result for the fixed image and the corresponding deformation field (Warp, third column). The first column shows the deformation field obtained in the negative direction (-Warp) and the corresponding deformed moving image. The second column shows the deformation field obtained in the half-positive direction (1/2 Warp) and the corresponding deformation moving image. The fourth column shows the deformation field obtained in the twice position direction (2 Warp) and the corresponding deformed moving image.

**Supplementary Fig. 2: The registration results of the fixed image to the standard brain atlas.**

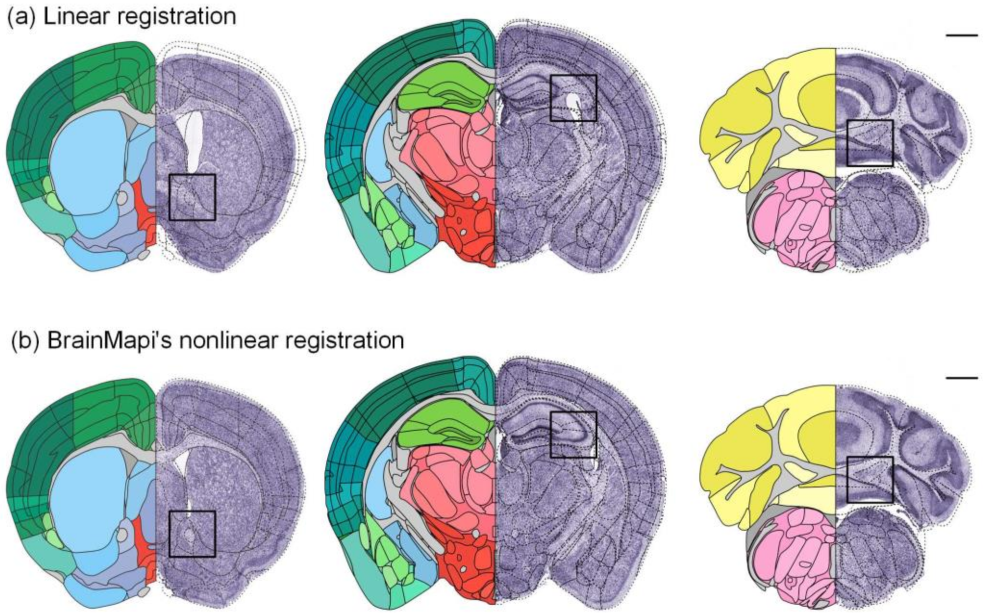


The 10 μm thick projections of three coronal sections. The left part of the images shows the Allen CCF v3, and the right part of the images shows the black dotted line of the Allen CCF v3 superimposed on the image. **(a)** The linear registration results for the Allen CCF v3 brain atlas. **(b)** The nonlinear registration results for the Allen CCF v3 brain atlas using the BrainMapi method. Scale bar: 1 mm. The region lines is from the Allen CCF v3, © 2004 Allen Institute for Brain Science. Allen Mouse Brain Atlas. Available from: atlas.brain-map.org.

**Supplementary Fig. 3: The architecture of the convolutional neural network.**

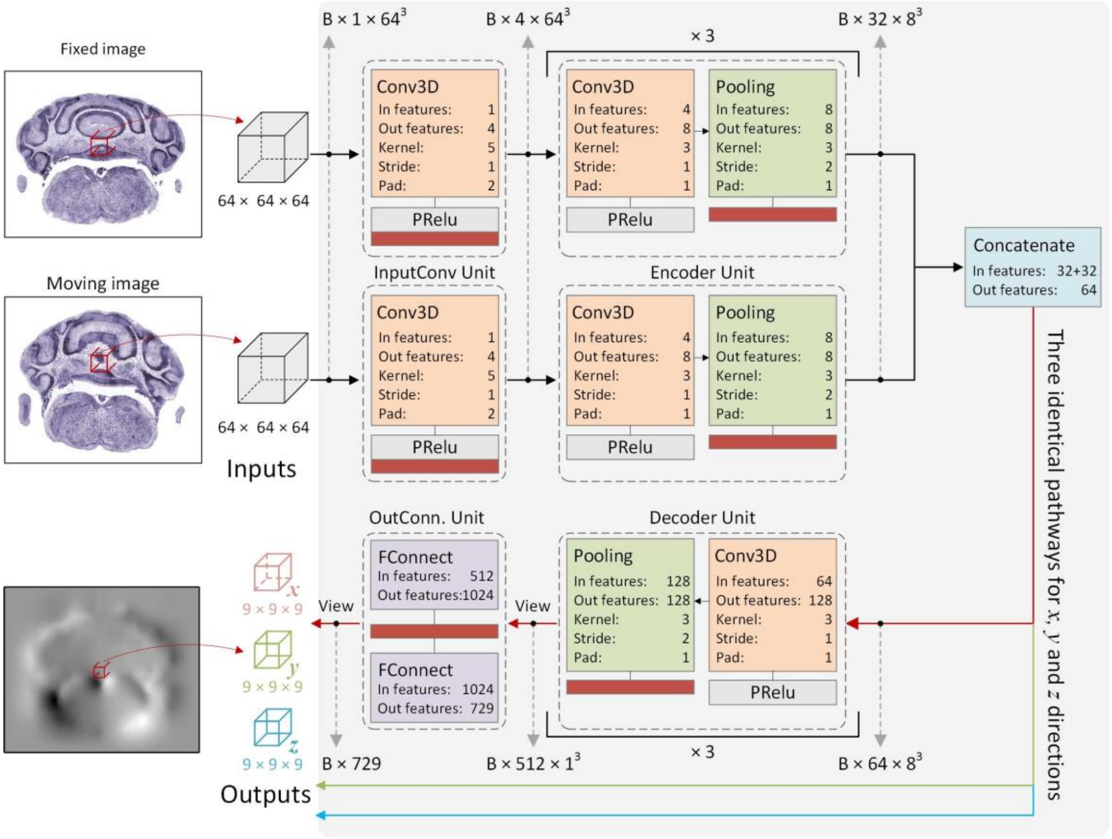


The variant patch-based network. The inputs are two 3D blocks from the fixed and moving images, respectively, and the outputs are three 3D patches corresponding to the displacement in the x, y and z directions. The network is composed of an InputConv Unit, Encoder Unit, Decoder Unit and OutConn Unit. Conv3D represents the 3D convolution layer, and Pooling indicates the pooling layer, Concatenate connects the two branches, and FConnect indicates the fully connected layer. The "kernel" represents the kernel size, "stride" indicates the step size, "pad" is the size of the edge zero padding during convolution or pooling, and B represents the batch size during training.

**Supplementary Fig. 4: The quantitative assessment before and after self-feedback.**

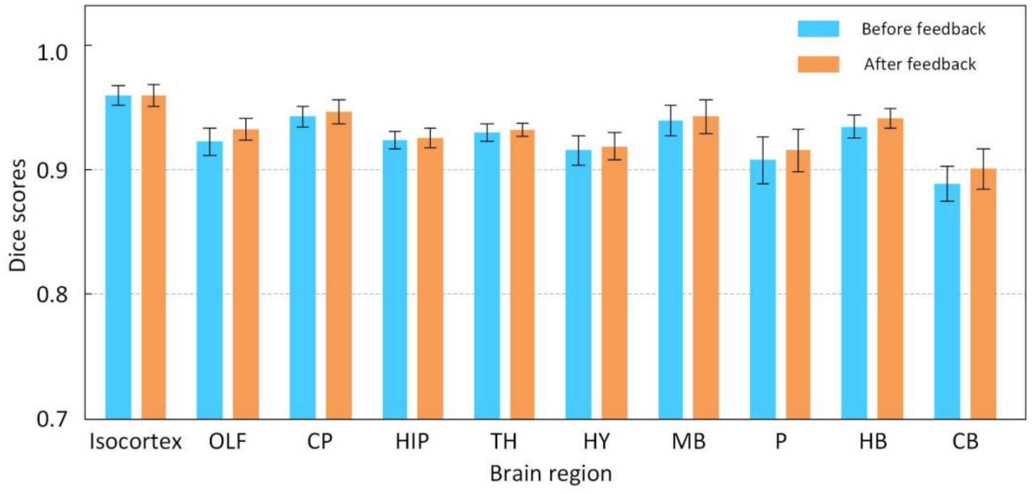


Mean Dice score comparisons of 10 brain regions before (blue) and after (orange) applying the self-feedback strategy.

**Supplementary Fig. 5: The quantitative assessment of the dual-hierarchical results.**

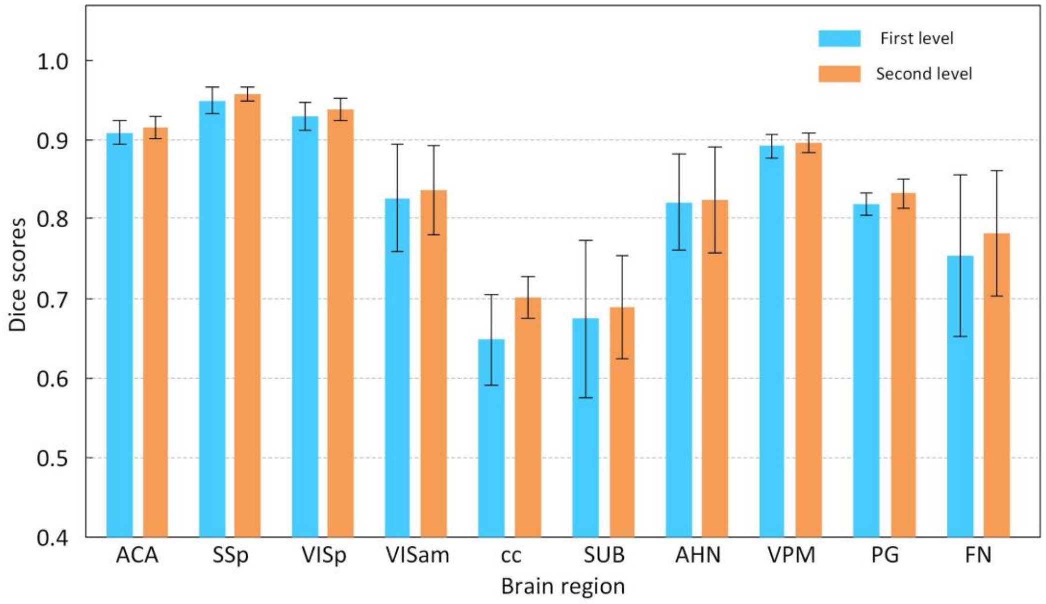


Mean Dice score comparisons of 10 smaller brain regions or nuclei in the first- and second-level.

**Supplementary Fig. 6: The registration results of different unsupervised methods.**

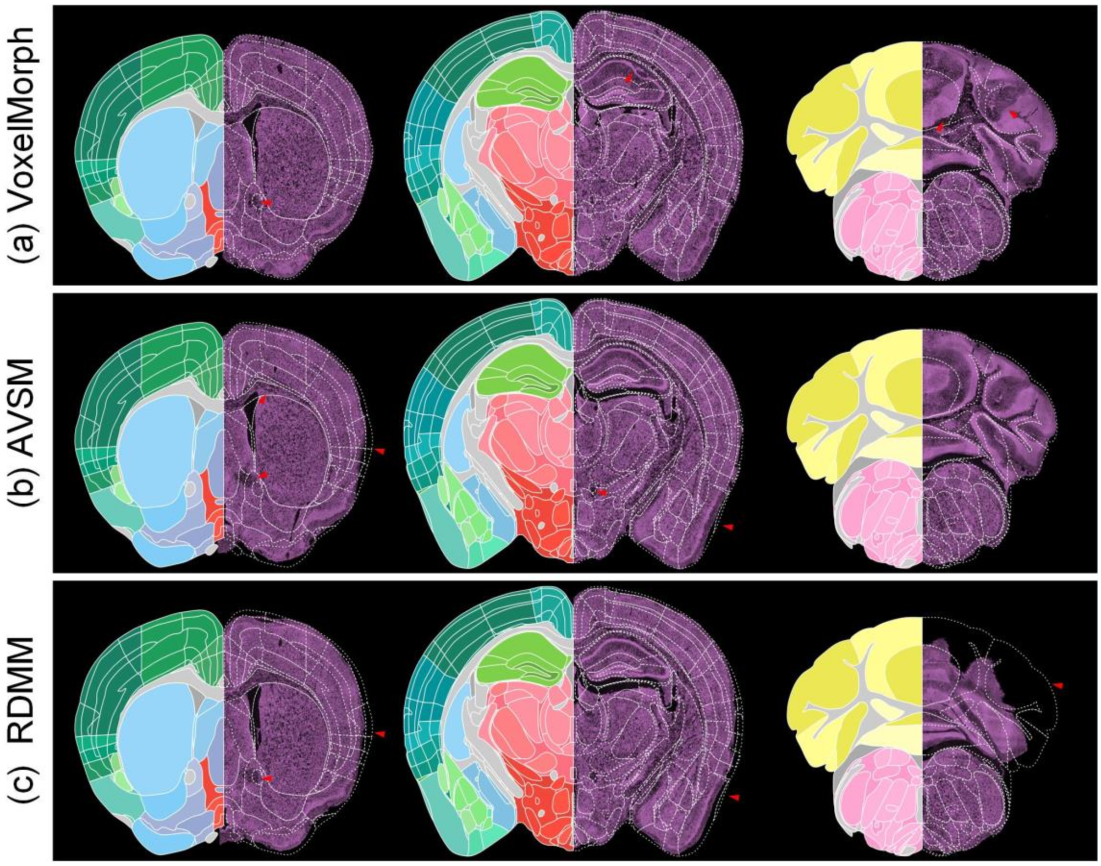


Three coronal sections. The left part of the image is the Allen CCF v3, and the right part of each image shows the white dotted line of the Allen CCF v3 superimposed on the registered image. The regions with inaccurate registration result are indicated with red arrows. **(a)** VoxelMorph. **(b)** AVSM. **(c)** RDMM. The region lines are from the Allen CCF v3, © 2004 Allen Institute for Brain Science. Allen Mouse Brain Atlas. Available from: atlas.brain-map.org.

**Supplementary Fig. 7: The colormap of Jacobian of the displacement field obtained by DeepMapi method.**

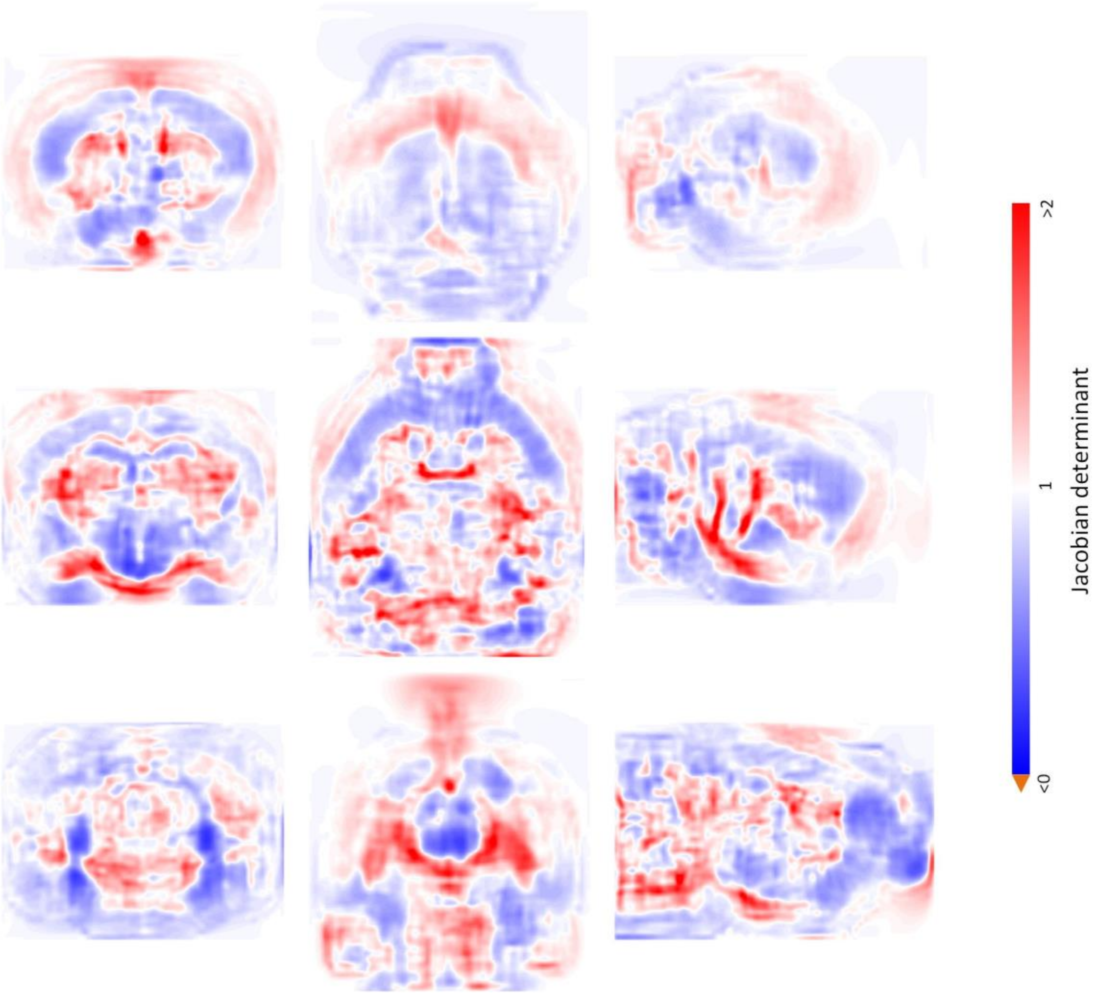


The nine anatomical sections of coronal, horizontal and sagittal, respectively. The color map of the Jacobian with singularities (folding) indicated in the brown (< 0), and as shown in the figure that there are no singularities by DeepMapi method.

**Supplementary Fig. 8: The colormap of Jacobian of the displacement field for Model 1-5, before/after self-feedback and with/without dual-hierarchical.**

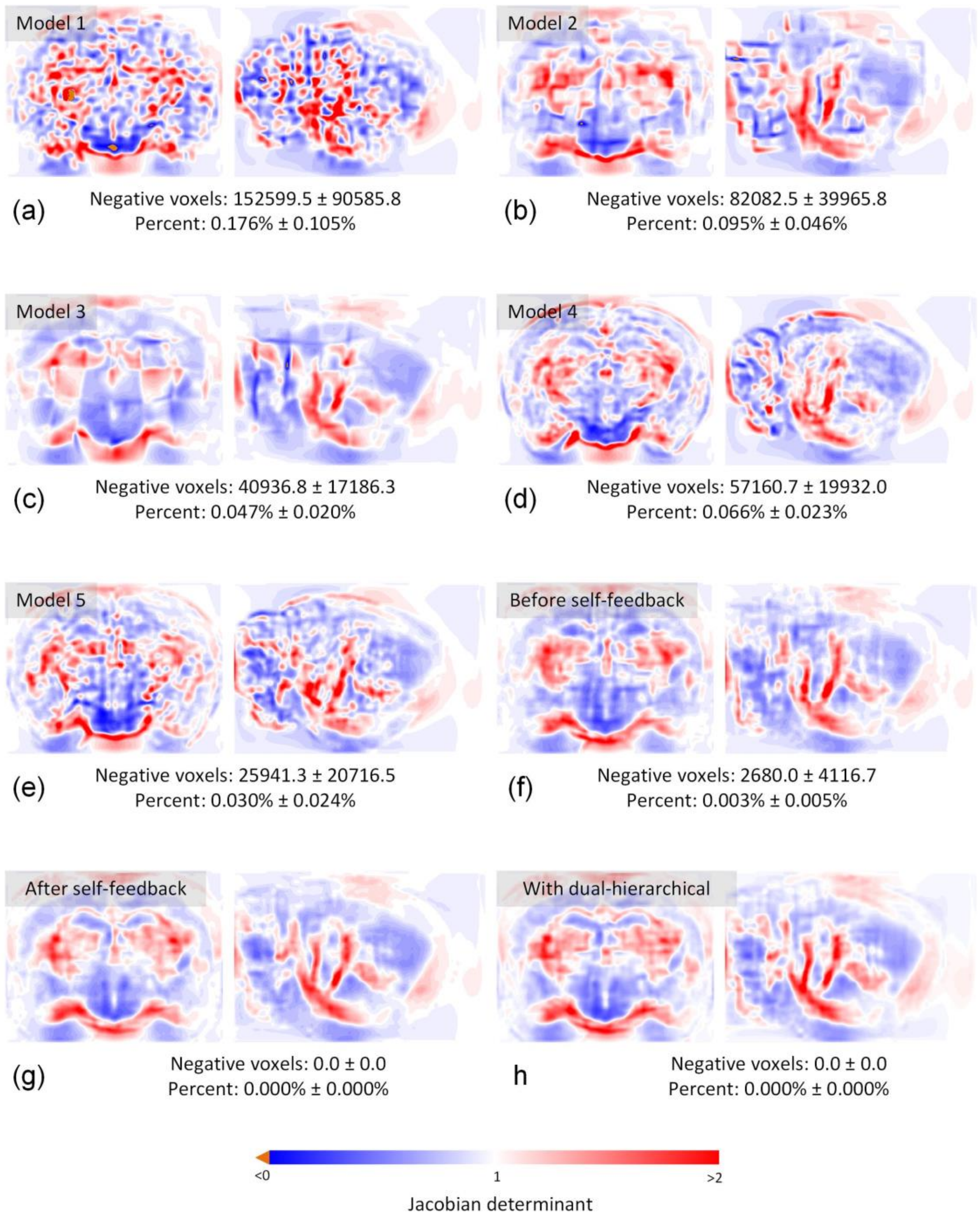


The anatomical sections of coronal and sagittal, respectively. The color map of the Jacobian with singularities (folding) indicated in the brown (< 0). **(a)** Model 1, **(b)** Model 2, **(c)** Model 3, **(d)** Model 4, **(e)** Model 5, **(f)** Before self-feedback, **(g)** After self-feedback, **(h)** With dual-hierarchical.

**Supplementary Table 1 Detail training information of six models.**

| | Model type | Input size | Output size | First feature number | E/D Coder number | Stride | Batch size |
|---|---|---|---|---|---|---|---|
| Model 1 | FCN | 16 | 16 | 32 | 2/2 | 32 | 64 |
| Model 2 | FCN | 32 | 32 | 16 | 3/3 | 32 | 16 |
| Model 3 | FCN | 64 | 64 | 8 | 3/3 | 64 | 4 |
| Model 4 | Patch | 16 | 3 | 32 | 2/2 | 16 | 64 |
| Model 5 | Patch | 32 | 5 | 16 | 3/2 | 32 | 32 |
| Model 6 | Patch | 64 | 9 | 4 | 3/3 | 32 | 64 |

**Supplementary Table 2 Comparisons of six models of loss value and running time.**

| Test model | Train loss/3pixels | Valid loss/3pixels | Training time | Predicted time |
|---|---|---|---|---|
| model 1 | 1.35 ± 0.05 | 7.41 ± 0.12 | 5d11h3m | 2m20s ± 1s |
| model 2 | 1.11 ± 0.03 | 5.21 ± 0.06 | 13d6h50m | 1m34s ± 1s |
| model 3 | 1.26 ± 0.04 | 3.45 ± 0.08 | **13d18h22m** | 1m39s ± 1s |
| model 4 | 0.97 ± 0.03 | 6.45 ± 0.03 | 4d11h44m | **21m4s ± 12s** |
| model 5 | 0.97 ± 0.05 | 4.99 ± 0.05 | 2d14h45m | 16m42s ± 5s |
| model 6 | **0.62 ± 0.03** | **3.17 ± 0.04** | 4d6h22m | 5m46s ± 2s |

**Supplementary Table 3 Mean absolute and mean Dice Scores of six models and P values of Wilcoxon signed-rank test among Model 6 and other five models.**

| Model | Model 1 | Model 2 | Model 3 | Model 4 | Model 5 | Model 6 |
|---|---|---|---|---|---|---|
| Mean absoute values (× 10 μm) | 2.490 ± 2.385 | 1.761 ± 1.733 | 1.521 ± 1.743 | 2.401 ± 2.208 | 1.861 ± 1.811 | **1.350 ± 1.475** |
| P values | 3.610e-41 | 8.112e-14 | 9.604e-04 | 3.078e-41 | 3.952e-18 | — |
| Mean dice scores | 0.885 ± 0.040 | 0.919 ± 0.025 | 0.924 ± 0.025 | 0.910 ± 0.031 | 0.915 ± 0.027 | **0.927 ± 0.022** |
| P values | 7.557e-10 | 4.537e-08 | 0.080 | 3.367e-09 | 9.068e-10 | — |

**Supplementary Table 4 Mean Dice Scores comparisons for ten large brain regions of six models.**

| Structures | Mean dice scores | | | | | |
|---|---|---|---|---|---|---|
| | model1 | model2 | model3 | model4 | model5 | model6 |
| Isocortex | 0.935 ± 0.015 | 0.958 ± 0.007 | 0.958 ± 0.011 | 0.953 ± 0.009 | 0.955 ± 0.008 | **0.960 ± 0.008** |
| OLF | 0.902 ± 0.019 | 0.918 ± 0.017 | **0.933 ± 0.014** | 0.913 ± 0.014 | 0.913 ± 0.012 | 0.923 ± 0.011 |
| CP | 0.903 ± 0.018 | 0.936 ± 0.013 | 0.939 ± 0.013 | 0.928 ± 0.019 | 0.935 ± 0.012 | **0.943 ± 0.008** |
| HIP | 0.850 ± 0.054 | 0.917 ± 0.014 | 0.914 ± 0.019 | 0.906 ± 0.025 | 0.909 ± 0.017 | **0.924 ± 0.007** |
| TH | 0.891 ± 0.023 | 0.917 ± 0.010 | 0.925 ± 0.013 | 0.910 ± 0.027 | 0.918 ± 0.015 | **0.930 ± 0.007** |
| HY | 0.861 ± 0.026 | 0.899 ± 0.013 | 0.905 ± 0.021 | 0.886 ± 0.031 | 0.889 ± 0.023 | **0.916 ± 0.012** |
| MB | 0.898 ± 0.027 | 0.931 ± 0.011 | 0.939 ± 0.009 | 0.924 ± 0.018 | 0.927 ± 0.014 | **0.940 ± 0.012** |
| P | 0.865 ± 0.029 | 0.899 ± 0.019 | 0.906 ± 0.017 | 0.886 ± 0.034 | 0.894 ± 0.026 | **0.908 ± 0.019** |
| HB | 0.912 ± 0.014 | 0.932 ± 0.012 | **0.937 ± 0.012** | 0.925 ± 0.019 | 0.930 ± 0.014 | 0.935 ± 0.009 |
| CB | 0.832 ± 0.033 | 0.882 ± 0.020 | 0.885 ± 0.017 | 0.874 ± 0.022 | 0.876 ± 0.017 | **0.889 ± 0.014** |

**Supplementary Table 5 Mean Dice Scores for ten brain regions before and after self-feedback.**

| Structures | Before feedback(Dice scores) | After feedback(Dice scores) |
|---|---|---|
| Isocortex | 0.960 ± 0.008 | **0.960 ± 0.009** |
| OLF | 0.923 ± 0.011 | **0.933 ± 0.009** |
| CP | 0.943 ± 0.008 | **0.947 ± 0.010** |
| HIP | 0.924 ± 0.007 | **0.926 ± 0.008** |
| TH | 0.930 ± 0.007 | **0.933 ± 0.005** |
| HY | 0.916 ± 0.012 | **0.919 ± 0.011** |
| MB | 0.940 ± 0.012 | **0.943 ± 0.014** |
| P | 0.908 ± 0.019 | **0.916 ± 0.017** |
| HB | 0.935 ± 0.009 | **0.942 ± 0.008** |
| CB | 0.889 ± 0.014 | **0.901 ± 0.016** |

**Supplementary Table 6 Mean Dice Scores for ten brain regions or nucleus in the first- and second-level.**

| Structures | First-hierarchy (Dice scores) | Second-hierarchy (Dice scores) |
|---|---|---|
| ACA | 0.908 ± 0.015 | **0.915 ± 0.014** |
| SSp | 0.949 ± 0.016 | **0.957 ± 0.008** |
| VISp | 0.929 ± 0.017 | **0.937 ± 0.014** |
| VISam | 0.826 ± 0.067 | **0.836 ± 0.056** |
| cc | 0.648 ± 0.057 | **0.701 ± 0.026** |
| SUB | 0.674 ± 0.099 | **0.688 ± 0.065** |
| AHN | 0.821 ± 0.061 | **0.824 ± 0.067** |
| VPM | 0.892 ± 0.015 | **0.896 ± 0.012** |
| PG | 0.819 ± 0.014 | **0.832 ± 0.018** |
| FN | 0.753 ± 0.102 | **0.781 ± 0.079** |

**Supplementary Table 7 Mean Dice Scores for 140 brain regions or nucleus of Affine (Aff.), LD demons (LD-D.), SyN, VoxelMorph (V.M.), AVSM (AV.), RDMM (RD.), QuickSilver (Q.S.), VNet (VN.) and DeepMapi (D.M.) methods.**

| No. | Brain regions | Brain region full name | Traditional method | | | Unsupervised method | | | Supervised method | | |
|---|---|---|---|---|---|---|---|---|---|---|---|
| | | | Aff. | LD-D. | SyN | V.M. | AV. | RD. | Q.S. | VN. | D.M. |
| 1 | MOs | Secondary motor area | 0.695 | 0.796 | 0.910 | 0.878 | 0.659 | 0.823 | 0.894 | 0.902 | 0.932 |
| 2 | MOp | Primary motor area | 0.741 | 0.759 | 0.922 | 0.905 | 0.658 | 0.852 | 0.920 | 0.917 | 0.943 |
| 3 | AId | Agranular insular area, dorsal part | 0.673 | 0.644 | 0.841 | 0.825 | 0.611 | 0.633 | 0.873 | 0.897 | 0.915 |
| 4 | AIv | Agranular insular area, ventral part | 0.636 | 0.553 | 0.763 | 0.787 | 0.626 | 0.616 | 0.841 | 0.878 | 0.887 |
| 5 | AIp | Agranular insular area, posterior part | 0.683 | 0.662 | 0.848 | 0.858 | 0.746 | 0.776 | 0.895 | 0.882 | 0.922 |
| 6 | ORBl | Orbital area, lateral part | 0.545 | 0.658 | 0.814 | 0.748 | 0.391 | 0.602 | 0.793 | 0.861 | 0.871 |
| 7 | ORBm | Orbital area, medial part | 0.375 | 0.591 | 0.761 | 0.644 | 0.126 | 0.417 | 0.729 | 0.802 | 0.719 |
| 8 | ORBvl | Orbital area, ventrolateral part | 0.456 | 0.560 | 0.802 | 0.668 | 0.183 | 0.490 | 0.724 | 0.813 | 0.768 |
| 9 | ACAv | Anterior cingulate area, ventral part | 0.544 | 0.753 | 0.880 | 0.796 | 0.661 | 0.787 | 0.780 | 0.806 | 0.875 |
| 10 | ACAd | Anterior cingulate area, dorsal part | 0.619 | 0.640 | 0.849 | 0.749 | 0.654 | 0.734 | 0.784 | 0.823 | 0.884 |
| 11 | PL | Prelimbic area | 0.547 | 0.696 | 0.852 | 0.760 | 0.334 | 0.676 | 0.803 | 0.838 | 0.866 |
| 12 | ILA | Infralimbic area | 0.081 | 0.586 | 0.822 | 0.643 | 0.212 | 0.509 | 0.706 | 0.748 | 0.769 |
| 13 | GU | Gustatory areas | 0.674 | 0.519 | 0.844 | 0.848 | 0.676 | 0.647 | 0.895 | 0.882 | 0.933 |
| 14 | SSp | Primary somatosensory area | 0.812 | 0.883 | 0.942 | 0.933 | 0.837 | 0.898 | 0.939 | 0.938 | 0.960 |
| 15 | SSs | Supplemental somatosensory area | 0.787 | 0.803 | 0.906 | 0.903 | 0.772 | 0.817 | 0.933 | 0.927 | 0.951 |
| 16 | VISC | Visceral area | 0.728 | 0.638 | 0.833 | 0.849 | 0.693 | 0.722 | 0.891 | 0.859 | 0.916 |
| 17 | AUDd | Dorsal auditory area | 0.721 | 0.473 | 0.720 | 0.779 | 0.505 | 0.698 | 0.853 | 0.833 | 0.901 |

| | | | | | | | | | | | |
|---|---|---|---|---|---|---|---|---|---|---|---|
| 18 | AUDp | Primary auditory area | 0.810 | 0.639 | 0.832 | 0.854 | 0.727 | 0.742 | 0.888 | 0.870 | 0.935 |
| 19 | AUDv | Ventral auditory area | 0.759 | 0.537 | 0.788 | 0.803 | 0.613 | 0.679 | 0.842 | 0.807 | 0.902 |
| 20 | PERI | Perirhinal area | 0.522 | 0.373 | 0.609 | 0.662 | 0.390 | 0.435 | 0.667 | 0.704 | 0.799 |
| 21 | ECT | Ectorhinal area | 0.706 | 0.539 | 0.753 | 0.777 | 0.565 | 0.630 | 0.789 | 0.812 | 0.872 |
| 22 | TEa | Temporal association areas | 0.754 | 0.638 | 0.829 | 0.817 | 0.659 | 0.663 | 0.826 | 0.822 | 0.898 |
| 23 | RSPagl | Retrosplenial area, lateral agranular part | 0.486 | 0.641 | 0.763 | 0.676 | 0.673 | 0.586 | 0.737 | 0.723 | 0.851 |
| 24 | RSPv | Retrosplenial area, ventral part | 0.691 | 0.789 | 0.854 | 0.859 | 0.784 | 0.803 | 0.863 | 0.840 | 0.899 |
| 25 | RSPd | Retrosplenial area, dorsal part | 0.543 | 0.717 | 0.810 | 0.780 | 0.749 | 0.729 | 0.810 | 0.793 | 0.877 |
| 26 | VISpm | posteromedial visual area | 0.734 | 0.656 | 0.848 | 0.760 | 0.570 | 0.612 | 0.825 | 0.822 | 0.863 |
| 27 | VISp | Primary visual area | 0.775 | 0.852 | 0.909 | 0.878 | 0.839 | 0.849 | 0.896 | 0.892 | 0.938 |
| 28 | VISL | Lateral visual area | 0.705 | 0.604 | 0.815 | 0.801 | 0.689 | 0.765 | 0.815 | 0.792 | 0.882 |
| 29 | MOB | Main olfactory bulb | 0.636 | 0.849 | 0.882 | 0.859 | 0.441 | 0.534 | 0.893 | 0.909 | 0.903 |
| 30 | AOB | Accessory olfactory bulb | 0.330 | 0.523 | 0.677 | 0.575 | 0.005 | 0.281 | 0.620 | 0.677 | 0.681 |
| 31 | AON | Anterior olfactory nucleus | 0.606 | 0.748 | 0.843 | 0.811 | 0.391 | 0.638 | 0.828 | 0.871 | 0.872 |
| 32 | TT | Taenia tecta | 0.364 | 0.666 | 0.762 | 0.777 | 0.188 | 0.572 | 0.777 | 0.775 | 0.822 |
| 33 | DP | Dorsal peduncular area | 0.012 | 0.522 | 0.748 | 0.586 | 0.012 | 0.474 | 0.607 | 0.631 | 0.768 |
| 34 | PIR | Piriform area | 0.754 | 0.815 | 0.870 | 0.879 | 0.803 | 0.790 | 0.904 | 0.923 | 0.938 |
| 35 | NLOT | Nucleus of the lateral olfactory tract | 0.481 | 0.633 | 0.747 | 0.735 | 0.403 | 0.693 | 0.805 | 0.800 | 0.886 |
| 36 | COA | Cortical amygdalar area | 0.472 | 0.810 | 0.858 | 0.852 | 0.764 | 0.742 | 0.857 | 0.849 | 0.917 |
| 37 | PAA | Piriform-amygdalar area | 0.308 | 0.730 | 0.800 | 0.763 | 0.705 | 0.630 | 0.814 | 0.825 | 0.897 |
| 38 | CA | Ammon''s horn | 0.705 | 0.866 | 0.874 | 0.823 | 0.794 | 0.822 | 0.846 | 0.819 | 0.913 |
| 39 | DG | Dentate gyrus | 0.589 | 0.813 | 0.827 | 0.752 | 0.730 | 0.760 | 0.770 | 0.724 | 0.863 |

| | | | | | | | | | | | |
|---|---|---|---|---|---|---|---|---|---|---|---|
| 40 | ENT | Entorhinal area | 0.712 | 0.851 | 0.881 | 0.828 | 0.794 | 0.772 | 0.844 | 0.840 | 0.903 |
| 41 | PAR | Parasubiculum | 0.393 | 0.487 | 0.566 | 0.479 | 0.431 | 0.539 | 0.514 | 0.475 | 0.656 |
| 42 | POST | Postsubiculum | 0.441 | 0.696 | 0.736 | 0.648 | 0.616 | 0.684 | 0.721 | 0.702 | 0.798 |
| 43 | PRE | Presubiculum | 0.429 | 0.581 | 0.637 | 0.474 | 0.518 | 0.604 | 0.545 | 0.502 | 0.691 |
| 44 | SUB | Subiculum | 0.374 | 0.632 | 0.681 | 0.531 | 0.506 | 0.619 | 0.555 | 0.476 | 0.685 |
| 45 | CLA | Claustrum | 0.312 | 0.139 | 0.311 | 0.459 | 0.341 | 0.510 | 0.636 | 0.737 | 0.768 |
| 46 | EPd | Endopiriform nucleus, dorsal part | 0.497 | 0.397 | 0.522 | 0.599 | 0.478 | 0.581 | 0.689 | 0.789 | 0.829 |
| 47 | LA | Lateral amygdalar nucleus | 0.412 | 0.532 | 0.640 | 0.622 | 0.492 | 0.567 | 0.700 | 0.738 | 0.813 |
| 48 | BLAa | Basolateral amygdalar nucleus, anterior part | 0.547 | 0.628 | 0.748 | 0.735 | 0.450 | 0.703 | 0.767 | 0.782 | 0.870 |
| 49 | BLAp | Basolateral amygdalar nucleus, posterior part | 0.527 | 0.615 | 0.722 | 0.680 | 0.528 | 0.628 | 0.707 | 0.768 | 0.854 |
| 50 | BLAv | Basolateral amygdalar nucleus, ventral part | 0.458 | 0.631 | 0.783 | 0.709 | 0.564 | 0.682 | 0.732 | 0.816 | 0.883 |
| 51 | BMA | Basomedial amygdalar nucleus | 0.573 | 0.666 | 0.780 | 0.767 | 0.634 | 0.746 | 0.789 | 0.832 | 0.894 |
| 52 | PA | Posterior amygdalar nucleus | 0.464 | 0.607 | 0.675 | 0.663 | 0.512 | 0.550 | 0.668 | 0.696 | 0.811 |
| 53 | ACB | Nucleus accumbens | 0.646 | 0.723 | 0.832 | 0.841 | 0.569 | 0.826 | 0.850 | 0.857 | 0.898 |
| 54 | OT | Olfactory tubercle | 0.709 | 0.777 | 0.813 | 0.853 | 0.670 | 0.712 | 0.882 | 0.889 | 0.917 |
| 55 | LSX | Lateral septal complex | 0.376 | 0.798 | 0.861 | 0.830 | 0.611 | 0.766 | 0.836 | 0.831 | 0.889 |
| 56 | BA | Bed nucleus of the accessory olfactory tract | 0.023 | 0.488 | 0.569 | 0.542 | 0.195 | 0.441 | 0.634 | 0.508 | 0.722 |
| 57 | CEA | Central amygdalar nucleus | 0.663 | 0.662 | 0.790 | 0.802 | 0.649 | 0.769 | 0.824 | 0.827 | 0.882 |
| 58 | MEA | Medial amygdalar nucleus | 0.629 | 0.780 | 0.843 | 0.828 | 0.698 | 0.796 | 0.850 | 0.847 | 0.897 |
| 59 | IA | Intercalated amygdalar nucleus | 0.277 | 0.350 | 0.559 | 0.517 | 0.189 | 0.476 | 0.589 | 0.583 | 0.741 |
| 60 | AAA | Anterior amygdalar area | 0.660 | 0.480 | 0.701 | 0.752 | 0.288 | 0.704 | 0.790 | 0.800 | 0.864 |
| 61 | GPe | Globus pallidus, external segment | 0.500 | 0.691 | 0.786 | 0.790 | 0.682 | 0.683 | 0.798 | 0.810 | 0.868 |

| | | | | | | | | | | | |
|---|---|---|---|---|---|---|---|---|---|---|---|
| 62 | BST | Bed nuclei of the stria terminalis | 0.419 | 0.726 | 0.785 | 0.740 | 0.529 | 0.700 | 0.722 | 0.752 | 0.823 |
| 63 | SI | Substantia innominata | 0.566 | 0.518 | 0.655 | 0.717 | 0.422 | 0.719 | 0.743 | 0.778 | 0.851 |
| 64 | MA | Magnocellular nucleus | 0.573 | 0.407 | 0.581 | 0.646 | 0.270 | 0.672 | 0.675 | 0.725 | 0.804 |
| 65 | NDB | Diagonal band nucleus | 0.377 | 0.554 | 0.690 | 0.665 | 0.345 | 0.668 | 0.702 | 0.695 | 0.807 |
| 66 | MS | Medial septal nucleus | 0.190 | 0.557 | 0.779 | 0.661 | 0.400 | 0.590 | 0.748 | 0.700 | 0.841 |
| 67 | SPFm | Subparafascicular nucleus, magnocellular part | 0.013 | 0.543 | 0.618 | 0.486 | 0.637 | 0.428 | 0.543 | 0.522 | 0.661 |
| 68 | SPFp | Subparafascicular nucleus, parvicellular part | 0.368 | 0.374 | 0.577 | 0.574 | 0.469 | 0.484 | 0.554 | 0.540 | 0.696 |
| 69 | SPA | Subparafascicular area | 0.063 | 0.667 | 0.757 | 0.562 | 0.693 | 0.543 | 0.559 | 0.596 | 0.694 |
| 70 | MG | Medial geniculate complex | 0.668 | 0.639 | 0.733 | 0.671 | 0.617 | 0.745 | 0.735 | 0.727 | 0.801 |
| 71 | LGd | Dorsal part of the lateral geniculate complex | 0.598 | 0.744 | 0.756 | 0.695 | 0.718 | 0.744 | 0.729 | 0.657 | 0.862 |
| 72 | PVT | Paraventricular nucleus of the thalamus | 0.334 | 0.724 | 0.809 | 0.713 | 0.605 | 0.492 | 0.692 | 0.695 | 0.809 |
| 73 | RE | Nucleus of reuniens | 0.375 | 0.632 | 0.732 | 0.580 | 0.615 | 0.557 | 0.573 | 0.616 | 0.807 |
| 74 | RH | Rhomboid nucleus | 0.251 | 0.375 | 0.568 | 0.313 | 0.549 | 0.454 | 0.352 | 0.399 | 0.602 |
| 75 | CM | Central medial nucleus of the thalamus | 0.235 | 0.569 | 0.620 | 0.556 | 0.376 | 0.359 | 0.598 | 0.615 | 0.688 |
| 76 | CL | Central lateral nucleus of the thalamus | 0.464 | 0.420 | 0.681 | 0.544 | 0.633 | 0.584 | 0.542 | 0.606 | 0.663 |
| 77 | PF | Parafascicular nucleus | 0.286 | 0.600 | 0.799 | 0.646 | 0.701 | 0.638 | 0.653 | 0.685 | 0.771 |
| 78 | IMD | Intermediodorsal nucleus of the thalamus | 0.400 | 0.505 | 0.714 | 0.564 | 0.672 | 0.541 | 0.594 | 0.657 | 0.740 |
| 79 | MD | Mediodorsal nucleus of thalamus | 0.573 | 0.744 | 0.881 | 0.770 | 0.814 | 0.765 | 0.776 | 0.808 | 0.865 |
| 80 | SMT | Submedial nucleus of the thalamus | 0.304 | 0.562 | 0.737 | 0.529 | 0.638 | 0.556 | 0.557 | 0.576 | 0.720 |
| 81 | LP | Lateral posterior nucleus of the thalamus | 0.583 | 0.705 | 0.762 | 0.707 | 0.687 | 0.732 | 0.699 | 0.741 | 0.838 |
| 82 | AV | Anteroventral nucleus of thalamus | 0.417 | 0.677 | 0.803 | 0.759 | 0.627 | 0.622 | 0.752 | 0.757 | 0.812 |
| 83 | AM | Anteromedial nucleus | 0.358 | 0.674 | 0.846 | 0.732 | 0.673 | 0.613 | 0.701 | 0.705 | 0.819 |

| | | | | | | | | | | | |
|---|---|---|---|---|---|---|---|---|---|---|---|
| 84 | LPO | Lateral preoptic area | 0.336 | 0.512 | 0.636 | 0.646 | 0.425 | 0.634 | 0.637 | 0.696 | 0.804 |
| 85 | LHA | Lateral hypothalamic area | 0.561 | 0.735 | 0.818 | 0.770 | 0.756 | 0.812 | 0.784 | 0.847 | 0.890 |
| 86 | RCH | Retrochiasmatic area | 0.117 | 0.504 | 0.541 | 0.524 | 0.459 | 0.432 | 0.507 | 0.534 | 0.706 |
| 87 | TU | Tuberal nucleus | 0.142 | 0.716 | 0.756 | 0.675 | 0.711 | 0.703 | 0.722 | 0.754 | 0.828 |
| 88 | ZI | Zona incerta | 0.571 | 0.674 | 0.755 | 0.712 | 0.701 | 0.669 | 0.720 | 0.748 | 0.830 |
| 89 | AHN | Anterior hypothalamic nucleus | 0.314 | 0.630 | 0.723 | 0.706 | 0.645 | 0.657 | 0.682 | 0.756 | 0.825 |
| 90 | MPN | Medial preoptic nucleus | 0.191 | 0.635 | 0.747 | 0.680 | 0.458 | 0.597 | 0.664 | 0.645 | 0.820 |
| 91 | VMH | Ventromedial hypothalamic nucleus | 0.317 | 0.743 | 0.774 | 0.616 | 0.781 | 0.746 | 0.674 | 0.759 | 0.844 |
| 92 | DMH | Dorsomedial nucleus of the hypothalamus | 0.302 | 0.651 | 0.778 | 0.576 | 0.696 | 0.624 | 0.582 | 0.712 | 0.808 |
| 93 | SBPV | Subparaventricular zone | 0.151 | 0.454 | 0.548 | 0.439 | 0.444 | 0.386 | 0.461 | 0.427 | 0.620 |
| 94 | PVH | Paraventricular hypothalamic nucleus | 0.303 | 0.584 | 0.632 | 0.463 | 0.501 | 0.393 | 0.481 | 0.562 | 0.762 |
| 95 | ARH | Arcuate hypothalamic nucleus | 0.070 | 0.720 | 0.727 | 0.576 | 0.711 | 0.625 | 0.618 | 0.605 | 0.787 |
| 96 | SCs | Superior colliculus, sensory related | 0.340 | 0.678 | 0.754 | 0.745 | 0.586 | 0.704 | 0.756 | 0.788 | 0.800 |
| 97 | IC | Inferior colliculus | 0.565 | 0.827 | 0.833 | 0.733 | 0.768 | 0.809 | 0.742 | 0.721 | 0.889 |
| 98 | NB | Nucleus of the brachium of the inferior colliculus | 0.292 | 0.538 | 0.599 | 0.378 | 0.399 | 0.492 | 0.505 | 0.561 | 0.643 |
| 99 | CUN | Cuneiform nucleus | 0.437 | 0.698 | 0.799 | 0.620 | 0.563 | 0.675 | 0.595 | 0.604 | 0.793 |
| 100 | RR | Midbrain reticular nucleus, retrorubral area | 0.454 | 0.582 | 0.644 | 0.637 | 0.588 | 0.622 | 0.669 | 0.647 | 0.770 |
| 101 | MRN | Midbrain reticular nucleus | 0.744 | 0.775 | 0.847 | 0.815 | 0.785 | 0.792 | 0.817 | 0.838 | 0.882 |
| 102 | PAG | Periaqueductal gray | 0.715 | 0.839 | 0.881 | 0.824 | 0.817 | 0.825 | 0.818 | 0.846 | 0.908 |
| 103 | VTA | Ventral tegmental area | 0.280 | 0.668 | 0.696 | 0.692 | 0.702 | 0.665 | 0.760 | 0.709 | 0.816 |
| 104 | SNr | Substantia nigra, reticular part | 0.531 | 0.773 | 0.815 | 0.782 | 0.761 | 0.772 | 0.812 | 0.789 | 0.877 |
| 105 | RN | Red nucleus | 0.697 | 0.804 | 0.851 | 0.832 | 0.804 | 0.797 | 0.844 | 0.859 | 0.885 |

| | | | | | | | | | | | |
|---|---|---|---|---|---|---|---|---|---|---|---|
| 106 | APN | Anterior pretectal nucleus | 0.631 | 0.771 | 0.851 | 0.786 | 0.786 | 0.824 | 0.792 | 0.800 | 0.864 |
| 107 | PPT | Posterior pretectal nucleus | 0.083 | 0.345 | 0.517 | 0.458 | 0.253 | 0.404 | 0.484 | 0.498 | 0.628 |
| 108 | SNc | Substantia nigra, compact part | 0.133 | 0.370 | 0.460 | 0.375 | 0.442 | 0.460 | 0.495 | 0.472 | 0.670 |
| 109 | PPN | Pedunculopontine nucleus | 0.707 | 0.748 | 0.842 | 0.798 | 0.759 | 0.753 | 0.779 | 0.772 | 0.867 |
| 110 | IPN | Interpeduncular nucleus | 0.420 | 0.775 | 0.810 | 0.772 | 0.706 | 0.680 | 0.820 | 0.832 | 0.863 |
| 111 | CLI | Central linear nucleus raphe | 0.263 | 0.679 | 0.702 | 0.688 | 0.642 | 0.617 | 0.707 | 0.711 | 0.803 |
| 112 | DR | Dorsal nucleus raphe | 0.553 | 0.691 | 0.767 | 0.669 | 0.574 | 0.615 | 0.605 | 0.673 | 0.772 |
| 113 | NLL | Nucleus of the lateral lemniscus | 0.444 | 0.654 | 0.752 | 0.676 | 0.626 | 0.641 | 0.696 | 0.671 | 0.787 |
| 114 | PSV | Principal sensory nucleus of the trigeminal | 0.481 | 0.727 | 0.811 | 0.753 | 0.751 | 0.768 | 0.770 | 0.766 | 0.843 |
| 115 | PB | Parabrachial nucleus | 0.490 | 0.706 | 0.793 | 0.675 | 0.614 | 0.662 | 0.661 | 0.618 | 0.815 |
| 116 | V | Motor nucleus of trigeminal | 0.622 | 0.629 | 0.788 | 0.708 | 0.749 | 0.768 | 0.750 | 0.757 | 0.841 |
| 117 | PRNc | Pontine reticular nucleus, caudal part | 0.716 | 0.705 | 0.835 | 0.776 | 0.823 | 0.803 | 0.847 | 0.884 | 0.893 |
| 118 | PCG | Pontine central gray | 0.402 | 0.627 | 0.707 | 0.611 | 0.491 | 0.564 | 0.598 | 0.603 | 0.756 |
| 119 | B | Barrington''s nucleus | 0.141 | 0.415 | 0.614 | 0.316 | 0.181 | 0.281 | 0.228 | 0.315 | 0.614 |
| 120 | CS | Superior central nucleus raphe | 0.669 | 0.700 | 0.812 | 0.768 | 0.757 | 0.741 | 0.835 | 0.854 | 0.887 |
| 121 | LDT | Laterodorsal tegmental nucleus | 0.362 | 0.618 | 0.729 | 0.565 | 0.554 | 0.591 | 0.550 | 0.521 | 0.759 |
| 122 | NI | Nucleus incertus | 0.467 | 0.502 | 0.612 | 0.527 | 0.384 | 0.558 | 0.643 | 0.726 | 0.782 |
| 123 | RPO | Nucleus raphe pontis | 0.617 | 0.567 | 0.650 | 0.609 | 0.593 | 0.641 | 0.717 | 0.701 | 0.776 |
| 124 | PG | Pontine gray | 0.555 | 0.813 | 0.822 | 0.743 | 0.679 | 0.707 | 0.792 | 0.782 | 0.835 |
| 125 | TRN | Tegmental reticular nucleus | 0.487 | 0.687 | 0.729 | 0.600 | 0.586 | 0.587 | 0.679 | 0.756 | 0.795 |
| 126 | PRNr | Pontine reticular nucleus | 0.748 | 0.765 | 0.871 | 0.830 | 0.822 | 0.814 | 0.862 | 0.890 | 0.912 |
| 127 | CN | Cochlear nuclei | 0.614 | 0.781 | 0.800 | 0.753 | 0.691 | 0.676 | 0.748 | 0.725 | 0.833 |

| | | | | | | | | | | | |
|---|---|---|---|---|---|---|---|---|---|---|---|
| 128 | DCN | Dorsal column nuclei | 0.654 | 0.362 | 0.780 | 0.682 | 0.302 | 0.045 | 0.748 | 0.727 | 0.826 |
| 129 | ECU | External cuneate nucleus | 0.353 | 0.492 | 0.598 | 0.590 | 0.338 | 0.001 | 0.640 | 0.615 | 0.746 |
| 130 | NTB | Nucleus of the trapezoid body | 0.284 | 0.587 | 0.630 | 0.543 | 0.666 | 0.509 | 0.666 | 0.751 | 0.699 |
| 131 | NTS | Nucleus of the solitary tract | 0.557 | 0.502 | 0.740 | 0.640 | 0.533 | 0.156 | 0.693 | 0.651 | 0.814 |
| 132 | GRN | Gigantocellular reticular nucleus | 0.766 | 0.672 | 0.815 | 0.813 | 0.814 | 0.658 | 0.842 | 0.860 | 0.890 |
| 133 | IRN | Intermediate reticular nucleus | 0.730 | 0.532 | 0.777 | 0.746 | 0.755 | 0.622 | 0.810 | 0.816 | 0.872 |
| 134 | PARN | Parvicellular reticular nucleus | 0.677 | 0.502 | 0.775 | 0.718 | 0.785 | 0.624 | 0.804 | 0.805 | 0.860 |
| 135 | MDRN | Medullary reticular nucleus | 0.807 | 0.495 | 0.811 | 0.773 | 0.514 | 0.148 | 0.862 | 0.855 | 0.887 |
| 136 | IO | Inferior olivary complex | 0.407 | 0.542 | 0.697 | 0.609 | 0.554 | 0.188 | 0.625 | 0.647 | 0.776 |
| 137 | LRN | Lateral reticular nucleus | 0.522 | 0.538 | 0.735 | 0.702 | 0.490 | 0.043 | 0.736 | 0.701 | 0.818 |
| 138 | MV | Medial vestibular nucleus | 0.740 | 0.817 | 0.892 | 0.828 | 0.759 | 0.691 | 0.837 | 0.820 | 0.893 |
| 139 | VERM | Vermal regions | 0.727 | 0.896 | 0.904 | 0.839 | 0.777 | 0.661 | 0.838 | 0.818 | 0.905 |
| 140 | HEM | Hemispheric regions | 0.721 | 0.900 | 0.904 | 0.858 | 0.782 | 0.565 | 0.835 | 0.832 | 0.902 |

**Supplementary Table 8 Mean Dice Scores of different methods and P values of Wilcoxon signed-rank test among DeepMapi and other methods.**

| Methods | | Mean dice scores | P values |
|---|---|---|---|
| | Affine | 0.493 ± 0.219 | 3.109e-116 |
| Traditional methods | LD Deomons | 0.637 ± 0.179 | 1.171e-111 |
| | SyN | 0.756 ± 0.127 | 2.217e-68 |
| | VoxelMorph | 0.698 ± 0.154 | 3.244e-112 |
| Unsupervised methods | AVSM | 0.582 ± 0.200 | 5.869e-113 |
| | RDMM | 0.623 ± 0.185 | 1.680e-112 |
| | QuickSilver | 0.728 ± 0.152 | 5.460e-92 |
| Supervised methods | VNet | 0.740 ± 0.151 | 2.382e-84 |
| | DeepMapi | 0.826 ± 0.091 | — |

**Supplementary Table 9 The comparison of self-feedback strategy with smaller step size, including the mean Dice Scores, P values and training time.**

| Sample strategy | Mean dice scores | P values | Training time approximate (days) |
|---|---|---|---|
| Self-feedback | 0.822 ± 0.101 | — | ~ 2 |
| 32 stride | 0.813 ± 0.101 | 9.8912e-13 | ~ 4 |
| 28 stride | 0.811 ± 0.102 | 1.9425e-09 | ~ 5 |
| 24 stride | 0.816 ± 0.099 | 3.1239e-06 | ~ 8 |
| 20 stride | 0.816 ± 0.100 | 1.5159e-04 | ~ 12 |
| 16 stride | 0.819 ± 0.096 | 0.019 | ~ 21 |
